# Discrete Transforms and Orthogonal Polynomials of (Anti)symmetric Multivariate Sine Functions

**DOI:** 10.3390/e20120938

**Published:** 2018-12-06

**Authors:** Adam Brus, Jiří Hrivnák, Lenka Motlochová

**Affiliations:** Department of Physics, Faculty of Nuclear Sciences and Physical Engineering, Czech Technical University in Prague, Břehová 7, 115 19 Prague 1, Czech Republic

**Keywords:** discrete multivariate sine transforms, orthogonal polynomials, cubature formulas

## Abstract

Sixteen types of the discrete multivariate transforms, induced by the multivariate antisymmetric and symmetric sine functions, are explicitly developed. Provided by the discrete transforms, inherent interpolation methods are formulated. The four generated classes of the corresponding orthogonal polynomials generalize the formation of the Chebyshev polynomials of the second and fourth kinds. Continuous orthogonality relations of the polynomials together with the inherent weight functions are deduced. Sixteen cubature rules, including the four Gaussian, are produced by the related discrete transforms. For the three-dimensional case, interpolation tests, unitary transform matrices and recursive algorithms for calculation of the polynomials are presented.

## 1. Introduction

The goal of this article is to develop discrete transforms of the multivariate symmetric and antisymmetric sine functions [1] together with the related Fourier interpolation and Chebyshev polynomial methods. The eight symmetric and eight antisymmetric discrete sine transforms form multivariate generalizations of the standard univariate discrete sine transforms [2] and correspond to their multivariate cosine counterparts from [3]. The cubature formulas of multivariate generalizations of the classical Chebyshev polynomials of the second and fourth kind are induced by the discrete transforms.

The multivariate (anti)symmetric trigonometric functions are constructed in [1] as trigonometric analogues of the Weyl orbit functions which are symmetrized or antisymetrized sums of exponential functions. The (anti)symmetrization is performed with respect to the Weyl group, which is a finite group generated by reflections and uniquely connected to a root system of a simple Lie algebra [4,5]. The continuous and discrete transforms together with interpolation tests of the bivariate cosine and sine cases are detailed in [6,7]. The specific connection between the Weyl orbit functions [4,5,8] and the (anti)symmetric trigonometric functions is deduced in [9]. For instance, since the Weyl group of simple Lie algebra $C_{n}$ is isomorphic to ${\left(Z/2Z\right)}^{n}⋊S_{n}$, the antisymetric sine functions coincide, up to a constant, with the antisymmetric Weyl orbit functions. The discrete transforms of the Weyl orbit functions on lattice fragments are induced by group theoretic arguments in [10,11,12,13]. On the other hand, the discrete transforms of the multivariate (anti)symmetric cosine transforms [3] are generated by their eight underlying standard univariate cosine transforms [2]. The eight univariate discrete sine transforms represent solutions of the discretized harmonic oscillator equation with distinct boundary conditions applied on the grid or mid-grid points [2]. These boundary conditions are inherited by the multivariate generalizations and combined with the behavior on the (anti)symmetrization boundaries determine overall properties of each discrete transform. The (anti)symmetric discrete sine transform developed from the discrete cosine transform of type I is derived in [1] and the antisymmetric discrete sine transforms are special cases of the transforms in [14]. However, the symmetric discrete transforms of types II–VIII in *n* variables have not been studied elsewhere. The multivariate (anti)symmetric trigonometric functions lead to the generalizations of the Chebyshev polynomials and the discrete transforms produce effective interpolation methods along with the related cubature integration formulas.

The four kinds of the classical Chebyshev polynomials serve as widely utilized orthogonal polynomials intertwined with powerful methods of numerical integration and approximation [15,16]. The (anti)symmetric cosine functions grant a multidimensional generalization of the Chebyshev polynomials of the first and third kinds [3], the present (anti)symmetric sine functions provide generalization of the second and fourth kinds firstly introduced in the present paper. The bivariate polynomials form special cases of analogues of the Jacobi polynomials [17]. The multivariate (anti)symmetric sine polynomials acquire essential characteristics from the (anti)symmetric sine functions and this connection grants required apparatus for generalization of efficient cubature formulas of the univariate Chebyshev polynomials. The fundamental goal of cubature formulas, replacing integration by optimal finite summation, is achieved via the suitable point sets of the generalized Chebyshev nodes [18]. Similarly to the classical univariate Chebyshev case, the resulting finite sum over the nodes equals exactly the approximated integral for polynomials up to a specific degree. Gaussian cubature formulas involve the lowest bound of the number of nodes and attain the maximal degree of precision [19,20,21]. Among the presented sixteen types of the symmetric sine cubature formulas four types are Gaussian which turn out to be special cases of formulas studied from different point of view in [22]. The other cubature formulas completing the set of integration formulas connected with the discrete multivariate sine transforms of types I–VIII are novel.

The successful interpolation tests for the 2D and 3D (anti)symmetric trigonometric functions are accomplished in [3,6,7]. The (anti)symmetric sine functions form solutions of the Laplace equation that satisfy a specific combination of the Dirichlet and von Neumann conditions on the boundaries of the fundamental domain [1]. The 2D and 3D (anti)symmetric sine functions as eigenfunctions of the discretized Laplace operator potentially represent solutions to lattice vibration models in solid state physics as well as foundation for description of the corresponding models in quantum field theory [23]. Boundary conditions of these models are determined by the boundary behavior of the multivariate discrete transforms and the spectral analysis provided by the developed transforms contributes to explicit solutions of the time evolution of the mechanical models. The approximation capability of the cubature formulas in the Weyl group setting that includes 2D cases of the (anti)symmetric trigonometric functions is successfully tested in [24]. The applications of Weyl orbit functions in image processing are developed in [25]. The potential physical applications of the studied cubature formulas encompass calculations in laser optics [26], stochastic dynamics [27], quantum dynamics [28], fluid flows [29], magnetostatic modeling [30], electromagnetic wave propagation [31], micromagnetic simulations [32], liquid crystal colloids [33] and porous materials [34,35].

The paper is organized as follows. In Section 2, the multivariate (anti)symmetric sine functions, their symmetry properties and continuous orthogonality are recalled. In Section 3, the sixteen types of the discrete (anti)symmetric sine transforms are listed and the interpolation method along, with the form of the unitary transform matrices, is presented. In Section 4, the multivariate generalization of the Chebyshev polynomials of the second and fourth kinds are introduced and the corresponding continuous orthogonality and cubature rules are deduced.

## 2. Multivariate (Anti)symmetric Sine Functions

### 2.1. Definitions and Symmetry Properties

The multivariate symmetric and antisymmetric generalizations of trigonometric functions are introduced in [1], symmetry properties of the (anti)symmetric discrete cosine transforms are detailed in [3]. The antisymmetric sine functions $\sin_{\lambda }^{-}\left(x\right)$ and the symmetric sine functions $\sin_{\lambda }^{+}\left(x\right)$, labeled by the parameter $\lambda =\left(\lambda_{1},\lambda_{2},\ldots ,\lambda_{n}\right)\in R^{n}$ and of the variable $x=\left(x_{1},x_{2},\ldots ,x_{n}\right)\in R^{n}$, are defined via the determinants and permanents of matrices with entries $\sin(\pi \lambda_{i}x_{j})$. Denoting the group of permutations by $S_{n}$ and the sign homomorphism on $S_{n}$ by sgn, the (anti)symmetric sine functions of several variables [1] are given explicitly by

(1) $$\begin{array}{cc} \sin_{\lambda }^{-}\left(x\right) & =\sum_{\sigma \in S_{n}}\mathrm{sgn}\left(\sigma \right)\sin\left(\pi \lambda_{\sigma (1)}x_{1}\right)\sin\left(\pi \lambda_{\sigma (2)}x_{2}\right)\cdots \sin\left(\pi \lambda_{\sigma (n)}x_{n}\right),\\ \sin_{\lambda }^{+}\left(x\right) & =\sum_{\sigma \in S_{n}}\sin\left(\pi \lambda_{\sigma (1)}x_{1}\right)\sin\left(\pi \lambda_{\sigma (2)}x_{2}\right)\cdots \sin\left(\pi \lambda_{\sigma (n)}x_{n}\right). \end{array}$$

The (anti)symmetric cosine functions [1] $\cos_{\lambda }^{\pm }\left(x\right)$ are for parameter $\lambda =\left(\lambda_{1},\lambda_{2},\ldots ,\lambda_{n}\right)\in R^{n}$ and variable $x=\left(x_{1},x_{2},\ldots ,x_{n}\right)\in R^{n}$ given similarly by the following formulas,

(2) $$\begin{array}{cc} \cos_{\lambda }^{-}\left(x\right) & =\sum_{\sigma \in S_{n}}\mathrm{sgn}\left(\sigma \right)\cos\left(\pi \lambda_{\sigma (1)}x_{1}\right)\cos\left(\pi \lambda_{\sigma (2)}x_{2}\right)\cdots \cos\left(\pi \lambda_{\sigma (n)}x_{n}\right),\\ \cos_{\lambda }^{+}\left(x\right) & =\sum_{\sigma \in S_{n}}\cos\left(\pi \lambda_{\sigma (1)}x_{1}\right)\cos\left(\pi \lambda_{\sigma (2)}x_{2}\right)\cdots \cos\left(\pi \lambda_{\sigma (n)}x_{n}\right). \end{array}$$

The (anti)symmetric sine functions posses several crucial symmetry properties [1]. Directly from definition (1), the multivariate sine functions $\sin_{\lambda }^{\pm }\left(x\right)$ are (anti)symmetric with respect to the action of a permutation $\sigma \in S_{n}$,

(3) $$\begin{array}{ccc} \sin_{\lambda }^{-}\left(\sigma \left(x\right)\right)=\mathrm{sgn}\left(\sigma \right)\sin_{\lambda }^{-}\left(x\right), & \; & \sin_{\sigma (\lambda )}^{-}\left(x\right)=\mathrm{sgn}\left(\sigma \right)\sin_{\lambda }^{-}\left(x\right),\\ \sin_{\lambda }^{+}\left(\sigma \left(x\right)\right)=\sin_{\lambda }^{+}\left(x\right), & \; & \sin_{\sigma (\lambda )}^{+}\left(x\right)=\sin_{\lambda }^{+}\left(x\right), \end{array}$$

where $\sigma \left(x\right)=\left(x_{\sigma (1)},x_{\sigma (2)},\cdots ,x_{\sigma (n)}\right)$ and $\sigma \left(\lambda \right)=\left(\lambda_{\sigma (1)},\lambda_{\sigma (2)},\cdots ,\lambda_{\sigma (n)}\right)$. Furthermore, the sine functions are anti–invariant with respect to sign alternations of both variables and parameters. For a change of sign $\tau_{i}$ of any *i*-th coordinate of the variable $x\in R^{n}$ or the parameter $\lambda \in R^{n}$,

(4) $$\begin{array}{cc} \tau_{i}\left(x_{1},\cdots ,x_{i},\cdots ,x_{n}\right) & =(x_{1},\cdots ,-x_{i},\cdots ,x_{n}),\\ \tau_{i}\left(\lambda_{1},\cdots ,\lambda_{i},\cdots ,\lambda_{n}\right) & =(\lambda_{1},\cdots ,-\lambda_{i},\cdots ,\lambda_{n}), \end{array}$$

it holds that

(5) $$\begin{array}{c} \sin_{\lambda }^{\pm }\left(\tau_{i}\left(x\right)\right)=-\sin_{\lambda }^{\pm }\left(x\right),\\ \sin_{\tau_{i}\left(\lambda \right)}^{\pm }\left(x\right)=-\sin_{\lambda }^{\pm }\left(x\right). \end{array}$$

Therefore, the functions $\sin_{\lambda }^{\pm }$ vanish if any coordinate of the variable $x_{i}$ or the parameter $\lambda_{i}$ are equal to zero.

Setting the ϱ-vector as

(6) $$\varrho =\left(\frac{1}{2},\frac{1}{2},\cdots ,\frac{1}{2}\right),$$

the functions $\sin_{k}^{\pm }$ and $\sin_{k-\varrho }^{\pm }$, $k\in Z^{n}$, admit additional symmetries with respect to multivariate integer shifts $t=\left(t_{1},t_{2},\cdots ,t_{n}\right)\in Z^{n}$ that stem from the periodicity of the univariate sine function,

(7) $$\begin{array}{cc} \sin_{k}^{\pm }\left(x+2t\right) & =\sin_{k}^{\pm }\left(x\right),\\ \sin_{k-\varrho }^{\pm }\left(x+2t\right) & ={\left(-1\right)}^{t_{1}+\cdots +t_{n}}\sin_{k-\varrho }^{\pm }\left(x\right). \end{array}$$

The sets of integer parameters $P_{1}^{\pm }$ are introduced as

(8) $$\begin{array}{cc} P_{1}^{+} & =\left\{\left(k_{1},k_{2},\cdots ,k_{n}\right)\in Z^{n}\;|\;k_{1}\geq k_{2}\geq \cdots \geq k_{n}\geq 1\right\},\\ P_{1}^{-} & =\left\{\left(k_{1},k_{2},\cdots ,k_{n}\right)\in Z^{n}\;|\;k_{1}>k_{2}>\cdots >k_{n}\geq 1\right\}. \end{array}$$

The relations (3) and (5) imply that it suffices to parametrize the functions $\sin_{k}^{\pm }$ and $\sin_{k-\varrho }^{\pm }$ only by the following values,

(9) $$\begin{array}{ccc} \sin_{k}^{+},\sin_{k-\varrho }^{+}: & \; & k\in P_{1}^{+},\\ \sin_{k}^{-},\sin_{k-\varrho }^{-}: & \; & k\in P_{1}^{-}. \end{array}$$

Due to relations (3), (5) and (7), the functions $\sin_{k}^{\pm }$ and $\sin_{k-\varrho }^{\pm }$ are restricted to the closure of the fundamental domain $F({\tilde{S}}_{n}^{\mathrm{aff}})$,

(10) $$F\left({\tilde{S}}_{n}^{\mathrm{aff}}\right)=\left\{\left(x_{1},x_{2},\cdots ,x_{n}\right)\in R^{n}\;|\;1\geq x_{1}\geq x_{2}\geq \ldots \geq x_{n}\geq 0\right\}.$$

Furthermore, it follows from the symmetry relations (3), (5) and the identity $\sin(\pi k_{i})=0$, $k_{i}\in Z$, that the functions $\sin_{k}^{\pm }$ and $\sin_{k-\varrho }^{\pm }$ are identically equal to zero on certain parts of the boundary of the domain $F({\tilde{S}}_{n}^{\mathrm{aff}})$. In particular, the following points are omitted from $F({\tilde{S}}_{n}^{\mathrm{aff}})$,

(11) $$\begin{array}{ccc} \sin_{k}^{-}\left(x\right): & \; & x_{i}=x_{i+1},i\in \left\{1,\ldots ,n-1\right\};\;x_{1}=1;\;x_{n}=0,\\ \sin_{k-\varrho }^{-}\left(x\right): & \; & x_{i}=x_{i+1},i\in \left\{1,\ldots ,n-1\right\};\;x_{n}=0,\\ \sin_{k}^{+}\left(x\right): & \; & x_{1}=1;\;x_{n}=0,\\ \sin_{k-\varrho }^{+}\left(x\right): & \; & x_{n}=0. \end{array}$$

In order to analyse polynomials of several variables in Section 4, four special cases of multivariate sine functions $\sin_{\lambda }^{\pm }$, labeled by the generalized ϱ-vectors

(12) $$\varrho_{2}^{-}=\left(n,n-1,\cdots ,1\right),\;\varrho_{2}^{+}=\left(1,1,\cdots ,1\right),\;\varrho_{4}^{-}=\left(n-\frac{1}{2},n-\frac{3}{2},\cdots ,\frac{1}{2}\right),\;\varrho_{4}^{+}=\varrho ,$$

are expressed in their product forms.

**Proposition** **1.** *Let k∈N be given by*

(13) $$k=\left\{\begin{array}{cc} \frac{n-1}{2} & \mathrm{for}\;n\;\mathrm{odd},\\ \frac{n}{2} & \mathrm{for}\;n\;\mathrm{even}. \end{array}\right.$$

*Then it holds that*

(14) $$\begin{array}{cccc} & \sin_{\varrho_{2}^{-}}^{-}\left(x_{1},\ldots ,x_{n}\right) & = & {\left(-1\right)}^{k}2^{n(n-1)}\prod_{i=1}^{n}\sin\left(\pi x_{i}\right)\prod_{1\leq i<j\leq n}\sin\left(\frac{\pi }{2}\left(x_{i}+x_{j}\right)\right)\sin\left(\frac{\pi }{2}\left(x_{i}-x_{j}\right)\right), \end{array}$$

(15) $$\begin{array}{cccc} & \sin_{\varrho_{2}^{+}}^{+}\left(x_{1},\ldots ,x_{n}\right) & = & n!\prod_{i=1}^{n}\sin\left(\pi x_{i}\right), \end{array}$$

(16) $$\begin{array}{cccc} & \sin_{\varrho_{4}^{-}}^{-}\left(x_{1},\ldots ,x_{n}\right) & = & {\left(-1\right)}^{k}2^{n(n-1)}\prod_{i=1}^{n}\sin\left(\frac{\pi }{2}x_{i}\right)\prod_{1\leq i<j\leq n}\sin\left(\frac{\pi }{2}\left(x_{i}+x_{j}\right)\right)\sin\left(\frac{\pi }{2}\left(x_{i}-x_{j}\right)\right), \end{array}$$

(17) $$\begin{array}{cccc} & \sin_{\varrho }^{+}\left(x_{1},\ldots ,x_{n}\right) & = & n!\prod_{i=1}^{n}\sin\left(\frac{\pi }{2}x_{i}\right). \end{array}$$

**Proof.**  The Formulas (15) and (17) follow directly from definition. The Equality (14) is derived in [3]. From the definition (1) and the symmetry property (3), the function $\sin_{\varrho_{4}^{-}}^{-}$ is given by

(18) $$\sin_{\varrho_{4}^{-}}^{-}\left(x_{1},\ldots ,x_{n}\right)={\left(-1\right)}^{k}\sin_{\left(\frac{1}{2},\frac{3}{2},\cdots ,n-\frac{1}{2}\right)}^{-}={\left(-1\right)}^{k}\det\left(\begin{array}{cccc} \sin\left(\frac{\pi }{2}x_{1}\right) & \sin\left(\frac{3\pi }{2}x_{1}\right) & \cdots & \sin\left(\frac{(2n-1)\pi }{2}x_{1}\right)\\ \sin\left(\frac{\pi }{2}x_{2}\right) & \sin\left(\frac{3\pi }{2}x_{2}\right) & \cdots & \sin\left(\frac{(2n-1)\pi }{2}x_{2}\right)\\ \vdots & \vdots & \ddots & \vdots \\ \sin\left(\frac{\pi }{2}x_{n}\right) & \sin\left(\frac{3\pi }{2}x_{n}\right) & \cdots & \sin\left(\frac{(2n-1)\pi }{2}x_{n}\right) \end{array}\right).$$

Basic properties of determinants together with the trigonometric identity for powers of the sine function

(19) sin((2m−1)θ)=(−1)m−122(m−1)sin2m−1(θ)−∑i=1m−1(−1)i2n−1isin(2n−1−2i)θ,m∈N,

and the power-reduction formula

(20) $$\cos\left(2\theta \right)=1-2\sin^{2}\left(\theta \right)$$

imply that the determinant (18) is of the following form,

(21) $$\sin_{\varrho_{4}^{-}}^{-}\left(x_{1},\ldots ,x_{n}\right)={\left(-1\right)}^{k}\det\left(\begin{array}{cccc} \sin\left(\frac{\pi }{2}x_{1}\right) & 2\sin\left(\frac{\pi }{2}x_{1}\right)\cos\left(\pi x_{1}\right) & \cdots & 2^{n-1}\sin\left(\frac{\pi }{2}x_{1}\right)\cos^{n-1}\left(\pi x_{1}\right)\\ \sin\left(\frac{\pi }{2}x_{2}\right) & 2\sin\left(\frac{\pi }{2}x_{1}\right)\cos\left(\pi x_{2}\right) & \cdots & 2^{n-1}\sin\left(\frac{\pi }{2}x_{1}\right)\cos^{n-1}\left(\pi x_{2}\right)\\ \vdots & \vdots & \ddots & \vdots \\ \sin\left(\frac{\pi }{2}x_{n}\right) & 2\sin\left(\frac{\pi }{2}x_{1}\right)\cos\left(\pi x_{n}\right) & \cdots & 2^{n-1}\sin\left(\frac{\pi }{2}x_{1}\right)\cos^{n-1}\left(\pi x_{n}\right) \end{array}\right).$$

The Formula (21) is rewritten as

(22) $$\sin_{\varrho_{4}^{-}}^{-}\left(x_{1},\ldots ,x_{n}\right)={\left(-1\right)}^{k}2^{\frac{n(n-1)}{2}}\prod_{i=1}^{n}\sin\left(\frac{\pi }{2}x_{i}\right)\det\left(\begin{array}{cccc} 1 & \cos(\pi x_{1}) & \cdots & \cos^{n-1}\left(\pi x_{1}\right)\\ 1 & \cos(\pi x_{2}) & \cdots & \cos^{n-1}\left(\pi x_{2}\right)\\ \vdots & \vdots & \ddots & \vdots \\ 1 & \cos(\pi x_{n}) & \cdots & \cos^{n-1}\left(\pi x_{n}\right) \end{array}\right).$$

Taking into account that the last determinant is of the Vandermonde type, it holds that

(23) $$\sin_{\varrho_{4}^{-}}^{-}\left(x_{1},\ldots ,x_{n}\right)={\left(-1\right)}^{k}2^{\frac{n(n-1)}{2}}\prod_{i=1}^{n}\sin\left(\frac{\pi }{2}x_{i}\right)\prod_{1\leq i<j\leq n}\cos\left(\pi x_{i}\right)-\cos\left(\pi x_{j}\right).$$

The sum-to-product trigonometric identity

(24) $$\cos\left(\pi x_{i}\right)-\cos\left(\pi x_{j}\right)=2\sin\left(\frac{\pi }{2}\left(x_{i}+x_{j}\right)\right)\sin\left(\frac{\pi }{2}\left(x_{i}-x_{j}\right)\right)$$

substituted in relation (23) yields Formula (16). □

Due to identities (14)–(17), the functions $\sin_{\varrho_{2}^{\pm }}^{\pm }$ and $\sin_{\varrho_{4}^{\pm }}^{-}$ vanish only on the parts of the boundary points of $F({\tilde{S}}_{n}^{\mathrm{aff}})$ specified by (11).

**Corollary** **1.** *The functions $\sin_{\varrho_{2}^{\pm }}^{\pm }$ and $\sin_{\varrho_{4}^{\pm }}^{-}$ are non-zero in the interior $F{\left({\tilde{S}}_{n}^{\mathrm{aff}}\right)}^{\circ }$ of the fundamental domain $F({\tilde{S}}_{n}^{\mathrm{aff}})$.*

**Figure 1:**
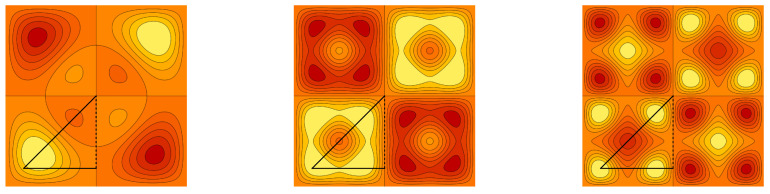
The contour plots of the symmetric trivariate sine function $\sin_{k}^{+}\left(x,y,1/5\right)$ with k=(2,1,1),(3,1,1) and (3,3,1). The border of the fundamental domain is depicted by the black line.

**Figure 2:**
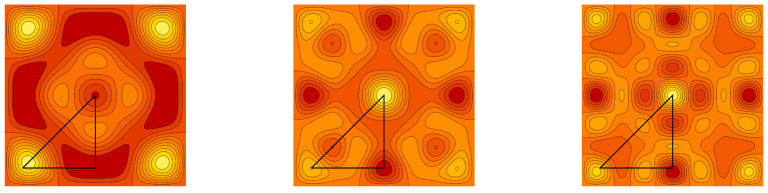
The contour plots of the symmetric trivariate sine function $\sin_{k-\varrho }^{+}\left(x,y,1/5\right)$ with k=(3,2,2),(4,2,2) and (4,4,2). The border of the fundamental domain is depicted by the black line.

**Figure 3:**
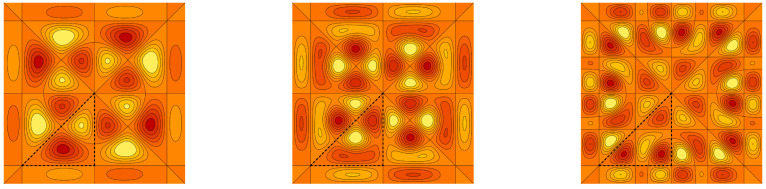
The contour plots of the antisymmetric trivariate sine function $\sin_{k}^{-}\left(x,y,1/5\right)$ with k=(4,2,1),(5,2,1),(5,4,1). The border of the fundamental domain is depicted by the black line.

**Figure 4:**
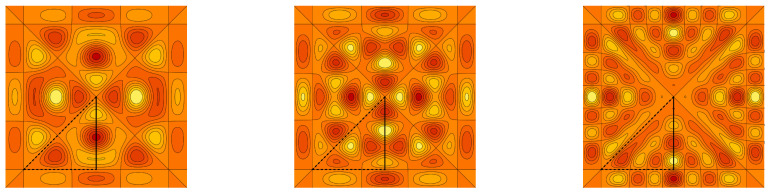
The contour plots of the antisymmetric trivariate sine function $\sin_{k-\varrho }^{-}\left(x,y,1/5\right)$ with k=(5,3,2),(6,3,2),(6,5,2). The border of the fundamental domain is depicted by the black line.

**Example** **1.** *Contour plots of the graph cuts z=1/5 of the symmetric trivariate sine function $\sin_{k}^{+}\left(x,y,z\right)$ and $\sin_{k-\varrho }^{+}\left(x,y,z\right)$ are for parameters k=(2,1,1),(3,1,1), (3,3,1) and k=(3,2,2),(4,2,2), (4,4,2) depicted in Figure 1 and Figure 2, respectively. Contour plots of the graph cuts z=1/5 of the antisymmetric trivariate sine function $\sin_{k}^{-}\left(x,y,z\right)$ and $\sin_{k-\varrho }^{-}\left(x,y,z\right)$ are for parameters k=(4,2,1),(5,2,1),(5,4,1) and k=(5,3,2),(6,3,2),(6,5,2) depicted in Figure 3 and Figure 4, respectively. The specific values of parameters are dispersed to visualize a wide range of different generalized trigonometric functions that possess common symmetry properties within each family. The plotting of the figures in this example, as well as plotting, numerical calculations and integrations in subsequent examples is performed by Wolfram Mathematica.*

### 2.2. Continuous Orthogonality

The antisymmetric and symmetric sine functions (9) are pairwise continuously orthogonal within each family when integrated over $F({\tilde{S}}_{n}^{\mathrm{aff}})$. Denoting the order of the stabilizer subgroup ${\mathrm{Stab}}_{S_{n}}\left(\lambda \right)$ of $S_{n}$ with respect to a point $\lambda \in R^{n}$ by $H_{\lambda }$,

(25) $$H_{\lambda }=\left|{\mathrm{Stab}}_{S_{n}}\left(\lambda \right)\right|,$$

the continuous orthogonality relations of the (anti)symmetric sine functions are given by

(26) $$\begin{array}{ccccc} \int_{F({\tilde{S}}_{n}^{\mathrm{aff}})}\sin_{k}^{-}\left(x\right)\sin_{k^{\prime }}^{-}\left(x\right)\;dx & \;=\; & 2^{-n}\delta_{kk^{\prime }}, & \; & k,k^{\prime }\in P_{1}^{-}, \end{array}$$

(27) $$\begin{array}{ccccc} \int_{F({\tilde{S}}_{n}^{\mathrm{aff}})}\sin_{k-\varrho }^{-}\left(x\right)\sin_{k^{\prime }-\varrho }^{-}\left(x\right)\;dx & \;=\; & 2^{-n}\delta_{kk^{\prime }}, & \; & k,k^{\prime }\in P_{1}^{-}, \end{array}$$

(28) $$\begin{array}{ccccc} \int_{F({\tilde{S}}_{n}^{\mathrm{aff}})}\sin_{k}^{+}\left(x\right)\sin_{k^{\prime }}^{+}\left(x\right)\;dx & \;=\; & 2^{-n}H_{k}\delta_{kk^{\prime }}, & \; & k,k^{\prime }\in P_{1}^{+}, \end{array}$$

(29) $$\begin{array}{ccccc} \int_{F({\tilde{S}}_{n}^{\mathrm{aff}})}\sin_{k-\varrho }^{+}\left(x\right)\sin_{k^{\prime }-\varrho }^{+}\left(x\right)\;dx & \;=\; & 2^{-n}H_{k}\delta_{kk^{\prime }}, & \; & k,k^{\prime }\in P_{1}^{+}, \end{array}$$

where $\delta_{kk^{\prime }}$ denotes the Kronecker delta.

The orthogonality relations (26) and (28) are deduced in [1] from the continuous orthogonality of univariate sine functions sin(πmθ), m∈N over the interval [0,1]. The remaining relations (27) and (29) follow similarly from the continuous orthogonality of the shifted sine functions sin(π(m−1/2)θ), m∈N,

(30) $$\int_{0}^{1}\sin\left(\pi \left(m-\frac{1}{2}\right)\theta \right)\sin\left(\pi \left(m^{\prime }-\frac{1}{2}\right)\theta \right)\;d\theta =\frac{1}{2}\delta_{mm^{\prime }},\;m,m^{\prime }\in N.$$

## 3. Discrete Transforms

The standard discrete sine transforms (DSTs) arise naturally from discretized solution of the harmonic oscillator equation with certain choices of boundary conditions [2]. The Dirichlet boundary condition is required at the beginning of the interval whereas the Neumann and Dirichlet boundary conditions are both allowed at the other end of the interval. Application of the boundary conditions at the grid or mid-grid points produces eight different transforms DST-I, ⋯, DST-VIII. The antisymmetric and symmetric generalizations of DSTs result in 16 various multivariate discrete transforms denoted by AMDST and SMDST respectively. The (anti-)symmetric multivariate sine transforms of type I are derived in [1] by employing DST-I. A similar method is used to complete the list of AMDST and SMDST.

In order to describe the generalized discrete transforms, two sets of labels $D_{1,N}$ and $D_{1,N,\varrho }$ are introduced for an arbitrary scaling factor N∈N,

(31) $$\begin{array}{cc} D_{1,N} & =\left\{\left(k_{1},k_{2},\cdots ,k_{n}\right)\in Z^{n}\;|\;N\geq k_{i}\geq 1,i=1,\cdots ,n\right\},\\ D_{1,N,\varrho } & =-\varrho +D_{1,N}=\left\{\left(k_{1}-\frac{1}{2},k_{2}-\frac{1}{2},\cdots ,k_{n}-\frac{1}{2}\right)\;|\;k_{i}\in Z^{n},\;N\geq k_{i}\geq 1,i=1,\cdots ,n\right\}. \end{array}$$

The normalization function *d* assigns to each label $k\in D_{1,N}$ the value $d_{k}$ determined by

(32) $$d_{k}=c_{k_{1}}c_{k_{2}}\cdots c_{k_{n}},\;c_{k_{i}}=\left\{\begin{array}{cc} \frac{1}{2} & \mathrm{if}\;k_{i}=N,\\ 1 & \mathrm{otherwise}. \end{array}\right.$$

Similarly, to each label $k\in D_{1,N,\varrho }$ is assigned the value ${\tilde{d}}_{k}$ equal to the previous discrete function evaluated at non-shifted point k+ϱ from $D_{1,N}$,

(33) $${\tilde{d}}_{k}=d_{k+\varrho }.$$

The generalized discrete transforms are developed on specific finite sets of points contained in $F({\tilde{S}}_{n}^{\mathrm{aff}})$. The point sets $C_{1,N}^{m}$ and $C_{1,N,\varrho }^{m}$ are subsets of two types of cubic lattices defined for four cases m∈{N+1,N,(2N+1)/2,(2N−1)/2} by

(34) $$C_{1,N}^{m}=\frac{1}{m}D_{1,N},\;C_{1,N,\varrho }^{m}=\frac{1}{m}D_{1,N,\varrho }.$$

The discrete weight function ε, defined for each point $s\in C_{1,N}^{m}$, is specified by the value of the function *d* on the point $ms\in D_{1,N}$,

(35) $$\varepsilon_{s}=d_{ms}.$$

The discrete function $\tilde{\varepsilon }$ is for each point $s\in C_{1,N,\varrho }^{m}$ given by

(36) $${\tilde{\varepsilon }}_{s}={\tilde{d}}_{ms}.$$

### 3.1. Antisymmetric Multivariate Discrete Sine Transforms

For an arbitrary scaling factor N∈N greater than or equal to *n*, AMDSTs express a discrete-valued function as a linear combination of antisymmetric sine functions. The functions $\sin_{k}^{-}$ are labeled by a finite set $D_{1,N}^{-}$ of labels in $P_{1}^{-}$ with coordinates not exceeding the value *N*,

(37) $$D_{1,N}^{-}=D_{1,N}\cap P_{1}^{-}=\left\{\left(k_{1},k_{2},\cdots ,k_{n}\right)\in Z^{n}\;|\;N\geq k_{1}>k_{2}>\ldots >k_{n}\geq 1\right\},$$

and by the label set $D_{1,N,\varrho }^{-}$ containing all labels of $D_{1,N}^{-}$ shifted by −ϱ,

(38) $$\begin{array}{cc} D_{1,N,\varrho }^{-} & =-\varrho +D_{1,N}^{-}=D_{1,N,\varrho }\cap \left\{-\varrho +P_{1}^{-}\right\}\\ & =\left\{\left(k_{1}-\frac{1}{2},k_{2}-\frac{1}{2},\cdots ,k_{n}-\frac{1}{2}\right)\;|\;k_{i}\in Z,\;N\geq k_{1}>k_{2}>\ldots >k_{n}\geq 1\right\}. \end{array}$$

In particular, Table 1 determines the finite set of labels $D_{N}^{★,-}$ and the corresponding finite set of points $F_{N}^{★,-}\subset F\left({\tilde{S}}_{n}^{\mathrm{aff}}\right)$, on which an expanded discrete function is evaluated, for each type ★∈{I,II,⋯,VIII} of AMDST. Each antisymmetric ★-type transform requires the inherent weights $\varepsilon^{★}$ and normalization coefficients $h^{★}$ listed also in Table 1. The antisymmetric sine functions labeled by $k,k^{\prime }\in D_{N}^{★,-}$ form an orthogonal basis of real-valued functions defined on the finite point set $F_{N}^{★,-}$ of each type,

(39) $$\sum_{s\in F_{N}^{★,-}}\varepsilon_{s}^{★}\sin_{k}^{-}\left(s\right)\sin_{k^{\prime }}^{-}\left(s\right)=h_{k}^{★}\delta_{kk^{\prime }}.$$

The discrete orthogonality (39) implies that any function $f:F_{N}^{★,-}\to R$ is expanded in terms of antisymmetric sine functions labeled by $k\in D_{N}^{★,-}$ as

(40) $$f\left(s\right)=\sum_{k\in D_{N}^{★,-}}A_{k}^{★}\sin_{k}^{-}\left(s\right),\;A_{k}^{★}=\frac{1}{h_{k}^{★}}\sum_{s\in F_{N}^{★,-}}\varepsilon_{s}^{★}f\left(s\right)\sin_{k}^{-}\left(s\right).$$

The eight types of AMDSTs specialize for n=1 to the corresponding standard DSTs.

**Remark** **1.** *The antisymmetric sine functions labeled by the parameters k of the form $\left(N+1,k_{2},\cdots ,k_{n}\right)$ and $\left(N+1/2,k_{2},\cdots ,k_{n}\right)$ are identically equal to zero for all points from the sets $F_{N}^{I,-}$ and $F_{N}^{\mathrm{VII},-}$, respectively. Therefore, the discrete orthogonality relations* (39) *for ★=I and ★=VII remain valid if either k or $k^{\prime }$, but not both, are of such form.*

### 3.2. Symmetric Multivariate Discrete Sine Transforms

For an arbitrary scaling factor N∈N, SMDSTs express a discrete-valued function as a linear combination of symmetric sine functions. The functions $\sin_{k}^{+}$ are labeled by a finite set $D_{1,N}^{+}$ of points in $P_{1}^{+}$ with coordinates not exceeding the value *N*,

(41) $$D_{1,N}^{+}=D_{1,N}\cap P_{1}^{+}=\left\{\left(k_{1},k_{2},\cdots ,k_{n}\right)\in Z^{n}\;|\;N\geq k_{1}\geq k_{2}\geq \ldots \geq k_{n}\geq 1\right\},$$

or by the set $D_{1,N,\varrho }^{+}$ containing all points of $D_{1,N}^{+}$ shifted by −ϱ,

(42) $$\begin{array}{cc} D_{1,N,\varrho }^{+} & =-\varrho +D_{1,N}^{+}=D_{1,N,\varrho }\cap \left\{-\varrho +P_{1}^{+}\right\}\\ & =\left\{\left(k_{1}-\frac{1}{2},k_{2}-\frac{1}{2},\cdots ,k_{n}-\frac{1}{2}\right)\;|\;N\geq k_{1}\geq k_{2}\geq \ldots \geq k_{n}\geq 1,k_{i}\in Z\right\}. \end{array}$$

In particular, Table 1 determines the finite set of labels $D_{N}^{★,+}$ and the corresponding finite set of points $F_{N}^{★,+}\subset F\left({\tilde{S}}_{n}^{\mathrm{aff}}\right)$, on which an expanded discrete function is evaluated, for each type ★∈{I,II,⋯,VIII} of SMDST. Besides the weights $\varepsilon^{★}$ and normalization coefficients $h^{★}$ from Table 1, the stabilizer function $H_{k}$, defined by (25), enters each symmetric transform. The symmetric sine functions, labeled by $k,k^{\prime }\in D_{N}^{★,+}$, form an orthogonal basis of real-valued functions defined on the finite point set $F_{N}^{★,+}$ of each type,

(43) $$\sum_{s\in F_{N}^{★,+}}\varepsilon_{s}^{★}H_{s}^{-1}\sin_{k}^{+}\left(s\right)\sin_{k^{\prime }}^{+}\left(s\right)=h_{k}^{★}H_{k}\delta_{kk^{\prime }}.$$

The discrete orthogonality (43) implies that any function $f:F_{N}^{★,+}\to R$ is expanded in terms of symmetric sine functions labeled by $k\in D_{N}^{★,+}$ as

(44) $$f\left(s\right)=\sum_{k\in D_{N}^{★,+}}A_{k}^{★}\sin_{k}^{+}\left(s\right),\;A_{k}^{★}=\frac{1}{h_{k}^{★}H_{k}}\sum_{s\in F_{N}^{★,+}}\varepsilon_{s}^{★}H_{s}^{-1}f\left(s\right)\sin_{k}^{+}\left(s\right).$$

The eight types of SMDSTs specialize for n=1 to the corresponding standard DSTs.

**Remark** **2.** *The symmetric sine functions, labeled by the parameters k of the form $\left(N+1,k_{2},\cdots ,k_{n}\right)$ and $\left(N+1/2,k_{2},\cdots ,k_{n}\right)$, are identically equal to zero for all points from the sets $F_{N}^{I,+}$ and $F_{N}^{\mathrm{VII},+}$, respectively. Therefore, the discrete orthogonality relations* (43) *for ★=I and ★=VII remain valid if k or $k^{\prime }$, but not both, are of such form.*

### 3.3. Interpolation by (Anti)symmetric Sine Functions

The developed formalism of discrete transforms provides solution of an interpolation problem formulated for a real-valued function over the fundamental domain $F({\tilde{S}}_{n}^{\mathrm{aff}})$. The interpolation problem for $f:F({\tilde{S}}_{n}^{\mathrm{aff}})\to R$ involves formation of an interpolation polynomial in terms of multivariate sine functions, labeled by parameters $k\in D_{N}^{★,\pm }$, ★∈{I,II,⋯,VIII},

(45) $$\psi_{N}^{★,\pm }\left(x\right)=\sum_{k\in D_{N}^{★,\mp }}A_{k}^{★}\sin_{k}^{\pm }\left(x\right).$$

The values of function *f* are required to coincide with the values of the interpolation polynomial on the corresponding finite set of points $F_{N}^{★,\pm }$,

(46) $$\psi_{N}^{★,\pm }\left(s\right)=f\left(s\right),\;s\in F_{N}^{★,\pm }.$$

Eight different types of antisymmetric interpolation polynomials $\psi_{N}^{★,-}$ and eight different types of symmetric interpolation polynomials $\psi_{N}^{★,+}$ are formed. Since the functions $\sin_{k}^{\pm }$ labeled by $k\in D_{N}^{★,\pm }$ form an orthogonal basis of all real-valued discrete functions on $F_{N}^{★,\pm }$, the coefficients $A_{k}^{★}$ are calculated by (40) and (44), respectively.

**Example** **2.** *For n=3, the following function f is chosen as a model function,*

(47) $$\begin{array}{cc} f(x,y,z) & =\exp\left(\frac{{\left(x-0,7\right)}^{2}+{\left(y-0,5\right)}^{2}+{\left(z-0,15\right)}^{2}}{0,005}+3\right)\\ & +\frac{1}{3}\exp\left(\frac{{\left(x-0,87\right)}^{2}+{\left(y-0,7\right)}^{2}+{\left(z-0,15\right)}^{2}}{0,005}+3\right). \end{array}$$

**Figure 5:**
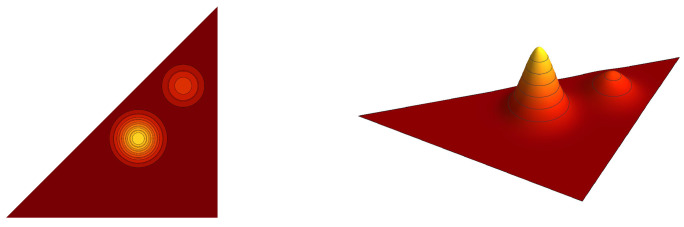
The graph cut of the model function (47) for fixed z=1/5.

**Figure 6:**
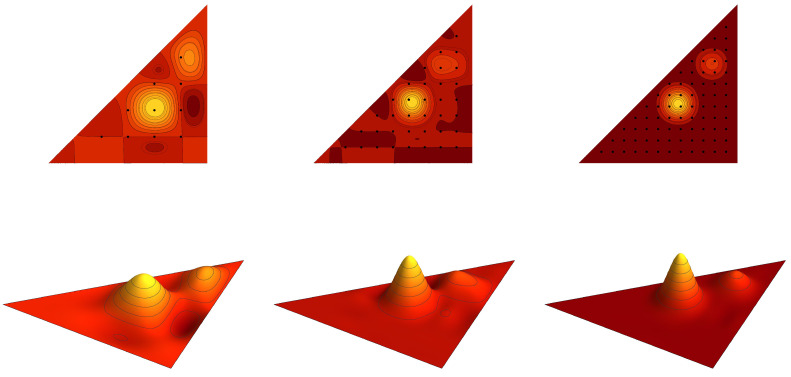
The antisymmetric interpolation polynomial $\psi_{N}^{\mathrm{VI},-}\left(x,y,1/5\right)$ of the model function (47) with N=7,12,17. The sets of points $F_{N}^{\mathrm{VI},-}$ are depicted as the black dots.

**Figure 7:**
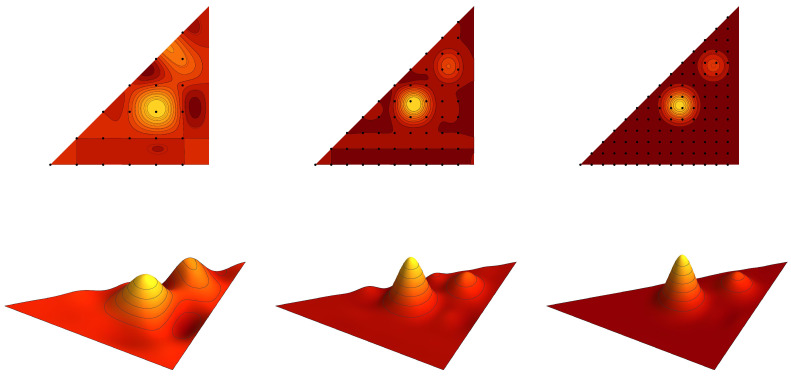
The symmetric interpolation polynomial $\psi_{N}^{\mathrm{VI},+}\left(x,y,1/5\right)$ of the model function (47) with N=7,12,17. The sets of points $F_{N}^{\mathrm{VI},+}$ are depicted as the black dots.

*The graph of the cut of f(x,y,z) by the plane z=1/5 in the fundamental domain $F({\tilde{S}}_{3}^{\mathrm{aff}})$ is plotted in Figure 5. The model function f is interpolated by the antisymmetric and symmetric polynomials of the sixth type $\psi_{N}^{\mathrm{VI},-}$ and $\psi_{N}^{\mathrm{VI},+}$, with N=7,12,17. The graph cuts of the interpolating polynomials are depicted in Figure 6 and Figure 7. Table 2 shows the integral error estimates for the polynomial approximations of the model function f by the antisymmetric and symmetric interpolation polynomials of type II, III, VI and VII for N=7,12,17,22,27.*

### 3.4. Matrices of the Normalized Discrete Trigonometric Transforms

The orthogonal matrices $S_{N}^{★,\pm }$, that correspond to the eight types of the normalized discrete sine transforms (40) and (44), are defined by relations

(48) $${\left(S_{N}^{★,+}\right)}_{k,s}=\sqrt{\frac{\varepsilon_{s}^{★}}{h_{k}^{★}H_{k}H_{s}}}\sin_{k}^{+}\left(s\right),\;{\left(S_{N}^{★,-}\right)}_{k,s}=\sqrt{\frac{\varepsilon_{s}^{★}}{h_{k}^{★}}}\sin_{k}^{-}\left(s\right).$$

The ordering inside the point and label sets is chosen as lexicographic.

**Example** **3.** *The orthogonal matrices $S_{3}^{\mathrm{VI},+}$ and $S_{5}^{\mathrm{VI},-}$ which realize the trivariate normalized transforms SMDST-VI and AMDST-VI are of the following explicit form,*

$$S_{3}^{\mathrm{VI},+}=\left(\begin{array}{cccccccccc} 0.035 & 0.137 & 0.309 & 0.400 & 0.110 & 0.350 & 0.556 & 0.198 & 0.446 & 0.206\\ 0.110 & 0.321 & 0.480 & 0.309 & 0.150 & 0.202 & -0.115 & -0.079 & -0.527 & -0.446\\ 0.198 & 0.385 & 0.321 & 0.137 & -0.079 & -0.404 & -0.293 & -0.337 & 0.115 & 0.556\\ 0.206 & 0.198 & 0.110 & 0.035 & -0.446 & -0.350 & -0.137 & 0.556 & 0.309 & -0.400\\ 0.137 & 0.293 & 0.115 & -0.556 & 0.321 & 0.404 & -0.337 & 0.385 & 0.079 & 0.198\\ 0.350 & 0.404 & -0.202 & -0.350 & 0.202 & -0.143 & 0.404 & -0.404 & 0.202 & -0.350\\ 0.446 & 0.079 & -0.150 & -0.110 & -0.527 & 0.202 & 0.321 & 0.115 & -0.480 & 0.309\\ 0.309 & 0.115 & -0.527 & 0.446 & 0.480 & -0.202 & -0.079 & 0.321 & -0.150 & 0.110\\ 0.556 & -0.337 & -0.079 & 0.198 & -0.115 & 0.404 & -0.385 & -0.293 & 0.321 & -0.137\\ 0.400 & -0.556 & 0.446 & -0.206 & 0.309 & -0.350 & 0.198 & 0.137 & -0.110 & 0.035 \end{array}\right),$$

$$S_{5}^{\mathrm{VI},-}=\left(\begin{array}{cccccccccc} -0.047 & -0.152 & -0.279 & -0.365 & -0.166 & -0.383 & -0.535 & -0.256 & -0.445 & -0.199\\ -0.166 & -0.365 & -0.383 & -0.279 & -0.256 & -0.199 & 0.047 & 0.152 & 0.535 & 0.445\\ -0.256 & -0.279 & -0.199 & -0.383 & 0.152 & 0.445 & 0.166 & 0.365 & -0.047 & -0.535\\ -0.199 & -0.047 & -0.256 & -0.166 & 0.445 & 0.152 & 0.383 & -0.535 & -0.279 & 0.365\\ -0.279 & -0.445 & -0.047 & 0.535 & -0.383 & -0.166 & 0.365 & -0.199 & -0.152 & -0.256\\ -0.535 & -0.256 & 0.365 & 0.152 & 0.047 & 0.279 & -0.445 & 0.166 & -0.199 & 0.383\\ -0.445 & 0.166 & -0.152 & 0.256 & 0.535 & -0.365 & -0.199 & -0.047 & 0.383 & -0.279\\ -0.365 & 0.199 & 0.535 & -0.445 & -0.279 & -0.047 & 0.152 & -0.383 & 0.256 & -0.166\\ -0.383 & 0.535 & -0.166 & -0.047 & -0.199 & -0.256 & 0.279 & 0.445 & -0.365 & 0.152\\ -0.152 & 0.383 & -0.445 & 0.199 & -0.365 & 0.535 & -0.256 & -0.279 & 0.166 & -0.047 \end{array}\right).$$

## 4. Chebyshev-Like Multivariate Orthogonal Polynomials

The Chebyshev polynomials of the first and third kind are generalized to multivariate orthogonal polynomials via the antisymmetric and symmetric cosine Function (2) in [3]. The multivariate generalizations of the Chebyshev polynomials of the second and fourth kind are built on the (anti)symmetric sine functions. Analogously to Chebyshev polynomials, the polynomial variables $X_{1},X_{2},\cdots ,X_{n}$ are associated with the functions of *n* variables given by

(49) $$X_{1}=\cos_{\left(1,0,\cdots ,0\right)}^{+},\;X_{2}=\cos_{\left(1,1,0\cdots ,0\right)}^{+},\;X_{3}=\cos_{\left(1,1,1,0,\cdots ,0\right)}^{+},\;\cdots ,\;X_{n}=\cos_{\left(1,1,\cdots ,1\right)}^{+}.$$

The common label set

(50) $$P^{+}=\left\{\left(k_{1},k_{2},\cdots ,k_{n}\right)\in Z^{n}\;|\;k_{1}\geq k_{2}\geq \cdots \geq k_{n}\geq 0\right\},$$

labels all four classes of orthogonal polynomials $P_{k}^{\mathrm{II},\pm },P_{k}^{\mathrm{IV},\pm }$, $k\in P^{+}$, in variables $X_{1},X_{2},\cdots ,X_{n}$ of degree $k_{1}$, induced for $x\in F{\left({\tilde{S}}_{n}^{\mathrm{aff}}\right)}^{\circ }$ by the following four rational functions,

(51) $$\begin{array}{ccc} P_{k}^{\mathrm{II},-}\left(X_{1}\left(x\right),X_{2}\left(x\right),\cdots ,X_{n}\left(x\right)\right)=\frac{\sin_{k+\varrho_{2}^{-}}^{-}\left(x\right)}{\sin_{\varrho_{2}^{-}}^{-}\left(x\right)}, & \; & P_{k}^{\mathrm{II},+}\left(X_{1}\left(x\right),X_{2}\left(x\right),\ldots ,X_{n}\left(x\right)\right)=\frac{\sin_{k+\varrho_{2}^{+}}^{+}\left(x\right)}{\sin_{\varrho_{2}^{+}}^{+}\left(x\right)},\\ P_{k}^{\mathrm{IV},-}\left(X_{1}\left(x\right),X_{2}\left(x\right),\cdots ,X_{n}\left(x\right)\right)=\frac{\sin_{k+\varrho_{4}^{-}}^{-}\left(x\right)}{\sin_{\varrho_{4}^{-}}^{-}\left(x\right)}, & \; & P_{k}^{\mathrm{IV},+}\left(X_{1}\left(x\right),X_{2}\left(x\right),\ldots ,X_{n}\left(x\right)\right)=\frac{\sin_{k+\varrho_{4}^{+}}^{+}\left(x\right)}{\sin_{\varrho_{4}^{+}}^{+}\left(x\right)}. \end{array}$$

Corollary 1 implies that the rational functions in (51) are well-defined on the interior of $F({\tilde{S}}_{n}^{\mathrm{aff}})$. The polynomials (51) are totally ordered by the lexicographic ordering > on $P^{+}$.

### 4.1. Recurrence Relations

The recursive construction of the polynomials is based on the generalized trigonometric identity that is derived from the classical product-to-sum trigonometric identity and valid for any $\lambda ,\mu \in R^{n}$,

(52) $$\sin_{\lambda }^{\pm }\left(x\right)\cos_{\mu }^{+}\left(x\right)=\frac{1}{2^{n}}\sum_{\sigma \in S_{n}}\sum_{\left\{\begin{array}{c} a_{i}=-1,1\\ i=1,\cdots ,n \end{array}\right\}}\sin_{\left(\lambda_{1}+a_{1}\mu_{\sigma (1)},\cdots ,\lambda_{n}+a_{n}\mu_{\sigma (n)}\right)}^{\pm }\left(x\right).$$

**Proposition** **2.** *The functions $P_{k}^{\mathrm{II},\pm }$, $P_{k}^{\mathrm{IV},\pm }$, $k\in P^{+}$ are polynomials in variables $X_{1},X_{2},\cdots ,X_{n}$, given by relation* (49), *of degree $k_{1}$.*

**Proof.**  Only the following special choices of parameters λ and μ appearing in (52) are considered,

$$\lambda \in \left\{\varrho_{2}^{\pm },\varrho_{4}^{\pm }\right\},\;\mu =k\in P^{+}.$$

Referring by minus sign to antisymmetric sign functions and by plus sign to symmetric sine functions, the labels from the right-side of (52) are denoted by $l^{\pm }$,

(53) $$l^{\pm }=\left(\lambda_{1}+a_{1}k_{\sigma (1)},\lambda_{2}+a_{2}k_{\sigma (2)},\cdots ,\lambda_{n}+a_{n}k_{\sigma (n)}\right),\;\sigma \in S_{n},\;a_{i}=-1,1.$$

All labels $l^{\pm }$ for which the generalized sine functions are identically equal to zero are excluded from consideration. Therefore, any label $l^{\pm }$ with at least one zero-valued coordinate and any label $l^{-}$ having at least two coordinates with the same absolute value are omitted.

For the remaining labels satisfy $l_{i}^{\pm }\neq 0$, there exists an alternation of signs τ of the negative coordinates such that all coordinates of $\tau (l^{\pm })$ are positive. Any sequence of (different) positive numbers can be rearranged by a certain permutation into a (decreasing) non-increasing sequence, i.e., there exists $\sigma^{\prime }\in S_{n}$ such that

(54) $$\begin{array}{c} {\left[\tau \left(l^{-}\right)\right]}_{\sigma^{\prime }\left(1\right)}>{\left[\tau \left(l^{-}\right)\right]}_{\sigma^{\prime }\left(2\right)}>\cdots >{\left[\tau \left(l^{-}\right)\right]}_{\sigma^{\prime }\left(n\right)}>0,\\ {\left[\tau \left(l^{+}\right)\right]}_{\sigma^{\prime }\left(1\right)}\geq {\left[\tau \left(l^{+}\right)\right]}_{\sigma^{\prime }\left(2\right)}\geq \cdots \geq {\left[\tau \left(l^{+}\right)\right]}_{\sigma^{\prime }\left(n\right)}>0. \end{array}$$

This implies that $\sigma^{\prime }\tau \left(l^{\pm }\right)$ lies in the set $\lambda +P^{+}$.

Setting

(55) $$\tilde{k}=\sigma^{\prime }\tau \left(l^{\pm }\right)-\lambda \in P^{+},$$

either $\tilde{k}=k$ or it holds that $k_{i}+\lambda_{i}>{\tilde{k}}_{i}+\lambda_{i}={\left[\sigma^{\prime }\tau \left(l^{\pm }\right)\right]}_{i}=\left|\lambda_{j}+a_{j}k_{\sigma (j)}\right|$ for the first *i* for which the coordinates of k+λ and $\tilde{k}+\lambda$ differ and some j∈{1,⋯,n}. Thus, *k* is lexicographically higher or equal to $\tilde{k}$. The equality is fulfilled if and only if $a_{i}=+1$ for all *i* for which $k_{i}>0$ and, in the case of the antisymmetric sine functions, if the additional condition that σ stabilizes *k* is satisfied. Considering the possible values of $a_{i}$ for which $k=\tilde{k}$, a function $T_{k}$ is defined by

(56) $$T_{k}=t_{k_{1}}t_{k_{2}}\cdots t_{k_{n}},\;t_{k_{i}}=\left\{\begin{array}{cc} 2 & \mathrm{if}\;k_{i}=0,\\ 1 & \mathrm{otherwise}. \end{array}\right.$$

Reflecting the possible values of σ, functions $H_{k}^{\pm }$ are introduced by

$$H_{k}^{-}=H_{k},\;H_{k}^{+}=n!.$$

By symmetry properties of multivariate sine functions, the Formula (52) is rewritten as

(57) $$\sin_{\lambda }^{\pm }\left(x\right)\cos_{k}^{+}\left(x\right)=\frac{1}{2^{n}}T_{k}H_{k}^{\pm }\sin_{k+\lambda }^{\pm }\left(x\right)+\sum_{k>\tilde{k}\in P^{+}}A_{\tilde{k}}\sin_{\tilde{k}+\lambda }^{\pm }\left(x\right),\;A_{\tilde{k}}\in R.$$

It follows from the Formula (57) that each function defined by (51) is built recurrently as certain linear combination of $\cos_{\tilde{k}}^{+}\left(x\right)$ parametrized by $\tilde{k}\leq k$ and that the coefficient of $\cos_{\tilde{k}}^{+}\left(x\right)$ with $\tilde{k}=k$ is non-zero. From Proposition 4.1 in [3], $\cos_{\tilde{k}}^{+}\left(x\right)$ is expressible as a polynomial function in variables $X_{1},X_{2},\cdots ,X_{n}$ of degree ${\tilde{k}}_{1}$. □

**Remark** **3.** *Proposition 2 implies that all the points for which the denominator is zero-valued are removable singularities. Therefore, it is possible to extend the domain of definition on the whole $F({\tilde{S}}_{n}^{\mathrm{aff}})$.*

Special choices of the labels in Formula (52) together with basic properties of multivariate sine functions suffice to deduce recurrence relations for the polynomials (51). In particular, the recurrence relations are deduced from the proof of Proposition 2, or by setting $\lambda =k\in P^{+}$ and equating successively μ to (1,0,⋯,0), (1,1,0,⋯,0), ⋯, (1,1,⋯,1) in (52). Thus, the recurrence algorithm is based on the following formulas,

(58) $$\begin{array}{cc} \sin_{k}^{\pm } & =\frac{2^{1}}{1!(n-1)!}\sin_{k-l_{1}}^{\pm }X_{1}-\sin_{k-2l_{1}}^{\pm }-\sum_{i=2}^{n}\sin_{k-l_{1}-l_{i}}^{\pm },\\ \sin_{k}^{\pm } & =\frac{2^{2}}{2!(n-2)!}\sin_{k-l_{1}-l_{2}}^{\pm }X_{2}-\sin_{k-2l_{1}-2l_{2}}^{\pm }-\sin_{k-2l_{1}}^{\pm }-\sin_{k-2l_{2}}^{\pm }\\ & -\sum_{i=3}^{n}\left(\sin_{k-l_{2}+l_{i}}^{\pm }+\sin_{k-2l_{1}-l_{2}-l_{i}}^{\pm }+\sin_{k-l_{2}-l_{i}}^{\pm }+\sin_{k-2l_{1}-l_{2}+l_{i}}^{\pm }\right)\\ & -\sum_{\overset{i,j=2}{i<j}}^{n}\left(\sin_{k-l_{1}-l_{2}+l_{i}+l_{j}}^{\pm }+\sin_{k-l_{1}-l_{2}-l_{i}-l_{j}}^{\pm }+\sin_{k-l_{1}-l_{2}+l_{i}-l_{j}}^{\pm }+\sin_{k-l_{1}-l_{2}-l_{i}+l_{j}}^{\pm }\right),\\ & \vdots \\ \sin_{k}^{\pm } & =\frac{2^{n}}{n!}\sin_{k-l_{1}-l_{2}-\ldots -l_{n}}^{\pm }X_{n}-\sum_{i}^{n}\sin_{k-2l_{i}}^{\pm }-\sum_{\overset{i,j=1}{i<j}}^{n}\sin_{k-2l_{i}-2l_{j}}^{\pm }-\ldots -\sin_{k-2l_{1}-2l_{2}-\ldots -2l_{n}}^{\pm }, \end{array}$$

where $l_{i}$ is a vector with *i*-th coordinate equal to 1 and others to 0.

**Example** **4.** *For n=3, the lowest polynomial of type $P_{k}^{\mathrm{II},+}$ is constant, $P_{\left(0,0,0\right)}^{\mathrm{II},+}=1$, and the first degree polynomials are given by*

(59) $$\;P_{\left(1,0,0\right)}^{\mathrm{II},+}=\frac{1}{3}X_{1},\;P_{\left(1,1,0\right)}^{\mathrm{II},+}=\frac{2}{3}X_{2},\;P_{\left(1,1,1\right)}^{\mathrm{II},+}=\frac{4}{3}X_{3}.$$

*The consecutive polynomials are then determined by the following recurrence relations.*

$$\begin{array}{cc} k_{1}\geq 2,\;k_{2}=k_{3}=0: & P_{\left(k_{1},0,0\right)}^{\mathrm{II},+}=P_{\left(k_{1}-1,0,0\right)}^{\mathrm{II},+}X_{1}-P_{\left(k_{1}-2,0,0\right)}^{\mathrm{II},+}-2P_{\left(k_{1}-1,1,0\right)}^{\mathrm{II},+}\\ k_{1}-1>k_{2}>k_{3}=0: & P_{\left(k_{1},k_{2},0\right)}^{\mathrm{II},+}=P_{\left(k_{1}-1,k_{2},0\right)}^{\mathrm{II},+}X_{1}-P_{\left(k_{1}-2,k_{2},0\right)}^{\mathrm{II},+}-P_{\left(k_{1}-1,k_{2}+1,0\right)}^{\mathrm{II},+}\\ & \;-P_{\left(k_{1}-1,k_{2}-1,0\right)}^{\mathrm{II},+}-P_{\left(k_{1}-1,k_{2},1\right)}^{\mathrm{II},+}\\ k_{1}-1>k_{2}=k_{3}>0: & P_{\left(k_{1},k_{2},k_{2}\right)}^{\mathrm{II},+}=P_{\left(k_{1}-1,k_{2},k_{2}\right)}^{\mathrm{II},+}X_{1}-P_{\left(k_{1}-2,k_{2},k_{2}\right)}^{\mathrm{II},+}\\ & \;-2P_{\left(k_{1}-1,k_{2}+1,k_{2}\right)}^{\mathrm{II},+}-2P_{\left(k_{1}-1,k_{2},k_{2}-1\right)}^{\mathrm{II},+}\\ k_{1}-1>k_{2}>k_{3}>0: & P_{\left(k_{1},k_{2},k_{3}\right)}^{\mathrm{II},+}=P_{\left(k_{1}-1,k_{2},k_{3}\right)}^{\mathrm{II},+}X_{1}-P_{\left(k_{1}-2,k_{2},k_{3}\right)}^{\mathrm{II},+}-P_{\left(k_{1}-1,k_{2}+1,k_{3}\right)}^{\mathrm{II},+}\\ & \;-P_{\left(k_{1}-1,k_{2}-1,k_{3}\right)}^{\mathrm{II},+}-P_{\left(k_{1}-1,k_{2},k_{3}+1\right)}^{\mathrm{II},+}-P_{\left(k_{1}-1,k_{2},k_{3}-1\right)}^{\mathrm{II},+}\\ k_{1}-1=k_{2}>k_{3}=0: & P_{\left(k_{1},k_{1}-1,0\right)}^{\mathrm{II},+}=\frac{1}{2}P_{\left(k_{1}-1,k_{1}-1,0\right)}^{\mathrm{II},+}X_{1}-P_{\left(k_{1}-1,k_{1}-2,0\right)}^{\mathrm{II},+}-\frac{1}{2}P_{\left(k_{1}-1,k_{1}-1,1\right)}^{\mathrm{II},+}\\ k_{1}-1=k_{2}>k_{3}>0: & P_{\left(k_{1},k_{1}-1,k_{3}\right)}^{\mathrm{II},+}=\frac{1}{2}P_{\left(k_{1}-1,k_{1}-1,k_{3}\right)}^{\mathrm{II},+}X_{1}-P_{\left(k_{1}-1,k_{1}-2,k_{3}\right)}^{\mathrm{II},+}\\ & \;-\frac{1}{2}P_{\left(k_{1}-1,k_{1}-1,k_{3}+1\right)}^{\mathrm{II},+}-\frac{1}{2}P_{\left(k_{1}-1,k_{1}-1,k_{3}-1\right)}^{\mathrm{II},+} \end{array}$$

$$\begin{array}{cc} k_{1}-1=k_{2}=k_{3}>0: & P_{\left(k_{1},k_{1}-1,k_{1}-1\right)}^{\mathrm{II},+}=\frac{1}{3}P_{\left(k_{1}-1,k_{1}-1,k_{1}-1\right)}^{\mathrm{II},+}X_{1}-P_{\left(k_{1}-1,k_{1}-1,k_{1}-2\right)}^{\mathrm{II},+}\\ k_{1}=k_{2}=2,\;k_{3}=0: & P_{\left(2,2,0\right)}^{\mathrm{II},+}=2P_{\left(1,1,0\right)}^{\mathrm{II},+}X_{2}-2P_{\left(1,0,0\right)}^{\mathrm{II},+}X_{1}-\frac{2}{3}P_{\left(1,1,1\right)}^{\mathrm{II},+}X_{1}+P_{\left(0,0,0\right)}^{\mathrm{II},+}+4P_{\left(1,1,0\right)}^{\mathrm{II},+}\\ k_{1}=k_{2}>2,\;k_{3}=0: & P_{\left(k_{1},k_{1},0\right)}^{\mathrm{II},+}=2P_{\left(k_{1}-1,k_{1}-1,0\right)}^{\mathrm{II},+}X_{2}-2P_{\left(k_{1}-1,k_{1}-2,0\right)}^{\mathrm{II},+}X_{1}-P_{\left(k_{1}-1,k_{1}-1,1\right)}^{\mathrm{II},+}X_{1}\\ & \;+P_{\left(k_{1}-2,k_{1}-2,0\right)}^{\mathrm{II},+}+3P_{\left(k_{1}-1,k_{1}-1,0\right)}^{\mathrm{II},+}+2P_{\left(k_{1}-1,k_{1}-2,1\right)}^{\mathrm{II},+}\\ & \;+2P_{\left(k_{1}-1,k_{1}-3,0\right)}^{\mathrm{II},+}+P_{\left(k_{1}-1,k_{1}-1,2\right)}^{\mathrm{II},+}\\ k_{1}=k_{2}>k_{3}+2>2: & P_{\left(k_{1},k_{1},k3\right)}^{\mathrm{II},+}=2P_{\left(k_{1}-1,k_{1}-1,k3\right)}^{\mathrm{II},+}X_{2}-2P_{\left(k_{1}-1,k_{1}-2,k3\right)}^{\mathrm{II},+}X_{1}\\ & \;-P_{\left(k_{1}-1,k_{1}-1,k_{3}+1\right)}^{\mathrm{II},+}X_{1}-P_{\left(k_{1}-1,k_{1}-1,k_{3}-1\right)}^{\mathrm{II},+}X_{1}+P_{\left(k_{1}-2,k_{1}-2,k_{3}\right)}^{\mathrm{II},+}\\ & \;+2P_{\left(k_{1}-1,k_{1}-2,k_{3}+1\right)}^{\mathrm{II},+}+2P_{\left(k_{1}-1,k_{1}-2,k_{3}-1\right)}^{\mathrm{II},+}+4P_{\left(k_{1}-1,k_{1}-1,k_{3}\right)}^{\mathrm{II},+}\\ & \;+2P_{\left(k_{1}-1,k_{1}-3,k_{3}\right)}^{\mathrm{II},+}+P_{\left(k_{1}-1,k_{1}-1,k_{3}+2\right)}^{\mathrm{II},+}+P_{\left(k_{1}-1,k_{1}-1,k_{3}-2\right)}^{\mathrm{II},+}\\ k_{1}=k_{2}=k_{3}+2=3: & P_{\left(3,3,1\right)}^{\mathrm{II},+}=2P_{\left(2,2,1\right)}^{\mathrm{II},+}X_{2}-2P_{\left(2,1,1\right)}^{\mathrm{II},+}X_{1}-\frac{2}{3}P_{\left(2,2,2\right)}^{\mathrm{II},+}X_{1}\\ & \;-P_{\left(2,2,0\right)}^{\mathrm{II},+}X_{1}+P_{\left(1,1,1\right)}^{\mathrm{II},+}+5P_{\left(2,2,1\right)}^{\mathrm{II},+}+4P_{\left(2,1,0\right)}^{\mathrm{II},+} \end{array}$$

(60) $$\begin{array}{cc} k_{1}=k_{2}=k_{3}+2>3: & P_{\left(k_{1},k_{1},k_{1}-2\right)}^{\mathrm{II},+}=2P_{\left(k_{1}-1,k_{1}-1,k_{1}-2\right)}^{\mathrm{II},+}X_{2}-2P_{\left(k_{1}-1,k_{1}-2,k_{1}-2\right)}^{\mathrm{II},+}X_{1}\\ & \;-\frac{2}{3}P_{\left(k_{1}-1,k_{1}-1,k_{1}-1\right)}^{\mathrm{II},+}X_{1}-P_{\left(k_{1}-1,k_{1}-1,k_{1}-3\right)}^{\mathrm{II},+}X_{1}+P_{\left(k_{1}-2,k_{1}-2,k_{1}-2\right)}^{\mathrm{II},+}\\ & \;+5P_{\left(k_{1}-1,k_{1}-1,k_{1}-2\right)}^{\mathrm{II},+}+4P_{\left(k_{1}-1,k_{1}-2,k_{1}-3\right)}^{\mathrm{II},+}+P_{\left(k_{1}-1,k_{1}-1,k_{1}-4\right)}^{\mathrm{II},+}\\ k_{1}=k_{2}=k_{3}+1=2: & P_{\left(2,2,1\right)}^{\mathrm{II},+}=\frac{2}{3}P_{\left(1,1,1\right)}^{\mathrm{II},+}X_{2}-P_{\left(1,1,0\right)}^{\mathrm{II},+}X_{1}+P_{\left(1,0,0\right)}^{\mathrm{II},+}+P_{\left(1,1,1\right)}^{\mathrm{II},+}\\ k_{1}=k_{2}=k_{3}+1>2: & P_{\left(k_{1},k_{1},k_{1}-1\right)}^{\mathrm{II},+}=\frac{2}{3}P_{\left(k_{1}-1,k_{1}-1,k_{1}-1\right)}^{\mathrm{II},+}X_{2}-P_{\left(k_{1}-1,k_{1}-1,k_{1}-2\right)}^{\mathrm{II},+}X_{1}\\ & \;+P_{\left(k_{1}-1,k_{1}-2,k_{1}-2\right)}^{\mathrm{II},+}+P_{\left(k_{1}-1,k_{1}-1,k_{1}-1\right)}^{\mathrm{II},+}+P_{\left(k_{1}-1,k_{1}-1,k_{1}-3\right)}^{\mathrm{II},+}\\ k_{1}=k_{2}=k_{3}=2: & P_{\left(2,2,2\right)}^{\mathrm{II},+}=\frac{4}{3}P_{\left(1,1,1\right)}^{\mathrm{II},+}X_{3}-6P_{\left(1,1,0\right)}^{\mathrm{II},+}X_{2}+3P_{\left(1,0,0\right)}^{\mathrm{II},+}X_{1}\\ & \;+2P_{\left(1,1,1\right)}^{\mathrm{II},+}X_{1}-P_{\left(0,0,0\right)}^{\mathrm{II},+}-6P_{\left(1,1,0\right)}^{\mathrm{II},+}\\ k_{1}=k_{2}=k_{3}=3: & P_{\left(3,3,3\right)}^{\mathrm{II},+}=\frac{4}{3}P_{\left(2,2,2\right)}^{\mathrm{II},+}X_{3}-6P_{\left(2,2,1\right)}^{\mathrm{II},+}X_{2}+3P_{\left(2,1,1\right)}^{\mathrm{II},+}X_{1}+2P_{\left(2,2,2\right)}^{\mathrm{II},+}X_{1}\\ & \;+3P_{\left(2,2,0\right)}^{\mathrm{II},+}X_{1}-P_{\left(1,1,1\right)}^{\mathrm{II},+}-9P_{\left(2,2,1\right)}^{\mathrm{II},+}-6P_{\left(2,1,0\right)}^{\mathrm{II},+}\\ k_{1}=k_{2}=k_{3}>3: & P_{\left(k_{1},k_{1},k_{1}\right)}^{\mathrm{II},+}=\frac{4}{3}P_{\left(k_{1}-1,k_{1}-1,k_{1}-1\right)}^{\mathrm{II},+}X_{3}-6P_{\left(k_{1}-1,k_{1}-1,k_{1}-2\right)}^{\mathrm{II},+}X_{2}\\ & \;+3P_{\left(k_{1}-1,k_{1}-2,k_{1}-2\right)}^{\mathrm{II},+}X_{1}+2P_{\left(k_{1}-1,k_{1}-1,k_{1}-1\right)}^{\mathrm{II},+}X_{1}\\ & \;+3P_{\left(k_{1}-1,k_{1}-1,k_{1}-3\right)}^{\mathrm{II},+}X_{1}-P_{\left(k_{1}-2,k_{1}-2,k_{1}-2\right)}^{\mathrm{II},+}-9P_{\left(k_{1}-1,k_{1}-1,k_{1}-2\right)}^{\mathrm{II},+}\\ & \;-6P_{\left(k_{1}-1,k_{1}-2,k_{1}-3\right)}^{\mathrm{II},+}-3P_{\left(k_{1}-1,k_{1}-1,k_{1}-4\right)}^{\mathrm{II},+}. \end{array}$$

*Similarly, the recurrence relations can be found for polynomials $P_{\left(k_{1},k_{2},k_{3}\right)}^{\mathrm{II},-}$ and $P_{\left(k_{1},k_{2},k_{3}\right)}^{\mathrm{IV},\pm }$. The polynomials $P_{\left(k_{1},k_{2},k_{3}\right)}^{\mathrm{II},\pm }$ and $P_{\left(k_{1},k_{2},k_{3}\right)}^{\mathrm{IV},\pm }$ of degree at most two are listed in Table 3, Table 4, Table 5 and Table 6.*

### 4.2. Continuous Orthogonality

The key notion that induces continuous orthogonality relations of the polynomials $P_{k}^{\mathrm{II},\pm }$ and $P_{k}^{\mathrm{IV},\pm }$ is the change of variables (49) in Formulas (26)–(28). The determinant of the Jacobian matrix

(61) $$J\left(x_{1},\ldots ,x_{n}\right)=\det\frac{\partial \left(X_{1},\ldots ,X_{n}\right)}{\partial \left(x_{1},\ldots ,x_{n}\right)}$$

for the change from the polynomial variables $\left(X_{1},\cdots ,X_{n}\right)$ to $\left(x_{1},\cdots ,x_{n}\right)$ is calculated in [3]. The absolute value of *J* is given by

(62) $$|J(x_{1},\cdots ,x_{n})\left|=c\right|\sin_{\varrho_{2}^{-}}^{-}\left(x_{1},x_{2},\cdots ,x_{n}\right)|,$$

with the constant *c* determined as

(63) $$c=\pi^{n}{\left(\frac{1}{2}\right)}^{\frac{n(n-1)}{2}}\left(\prod_{i=1}^{n}\left(n-i\right)!i!\right).$$

The absolute value $|J(x_{1},\cdots ,x_{n})|$ is shown in [3] to be expressible as a function J in the polynomial variables $\left(X_{1},\cdots ,X_{n}\right)$,

(64) $$J\left(X_{1},\cdots ,X_{n}\right)=\left|J(x_{1},\cdots ,x_{n})\right|.$$

Positivity of the Jacobian $J(X_{1}\left(x\right),\cdots ,X_{n}\left(x\right))>0$ is for all $x\in F{\left({\tilde{S}}_{n}^{\mathrm{aff}}\right)}^{\circ }$ guaranteed by Corollary 1.

In order to define the underlying weight functions, properties of the three additional products of multivariate sine functions $\sin_{\varrho_{2}^{+}}^{+}\left(x\right)\cdot \sin_{\varrho_{2}^{+}}^{+}\left(x\right)$, $\sin_{\varrho_{4}^{-}}^{-}\left(x\right)\cdot \sin_{\varrho_{4}^{-}}^{-}\left(x\right)$ and $\sin_{\varrho_{4}^{+}}^{+}\left(x\right)\cdot \sin_{\varrho_{4}^{+}}^{+}\left(x\right)$ are determined. Similarly to Formula (52), products of the (anti)symmetric sine functions are by a classical product-to-sum identity decomposed into a sum of the symmetric cosine functions. Denoting the number of positive $a_{i}$ by $\alpha (a_{1},\cdots ,a_{n})$, it holds that

(65) $$\begin{array}{ccc} \sin_{\lambda }^{-}\left(x\right)\sin_{\mu }^{-}\left(x\right) & = & \frac{1}{2^{n}}\sum_{\sigma \in S_{n}}\mathrm{sgn}\left(\sigma \right)\sum_{\left\{\begin{array}{c} a_{i}=-1,1\\ i=1,\cdots ,n \end{array}\right\}}{\left(-1\right)}^{\alpha (a_{1},\cdots ,a_{n})}\cos_{\left(\lambda_{1}+a_{1}\mu_{\sigma (1)},\cdots ,\lambda_{n}+a_{n}\mu_{\sigma (n)}\right)}^{+}\left(x\right),\\ \sin_{\lambda }^{+}\left(x\right)\sin_{\mu }^{+}\left(x\right) & = & \frac{1}{2^{n}}\sum_{\sigma \in S_{n}}\sum_{\left\{\begin{array}{c} a_{i}=-1,1\\ i=1,\cdots ,n \end{array}\right\}}{\left(-1\right)}^{\alpha (a_{1},\cdots ,a_{n})}\cos_{\left(\lambda_{1}+a_{1}\mu_{\sigma (1)},\cdots ,\lambda_{n}+a_{n}\mu_{\sigma (n)}\right)}^{+}\left(x\right). \end{array}$$

Therefore, all three products are expressed as polynomials in the variables $X_{1},\cdots ,X_{n}$,

(66) $$\begin{array}{cc} J^{\mathrm{II},+}\left(X_{1}\left(x\right),\ldots ,X_{n}\left(x\right)\right) & =\sin_{\varrho_{2}^{+}}^{+}\left(x\right)\sin_{\varrho_{2}^{+}}^{+}\left(x\right),\\ J^{\mathrm{IV},-}\left(X_{1}\left(x\right),\ldots ,X_{n}\left(x\right)\right) & =\sin_{\varrho_{4}^{-}}^{-}\left(x\right)\sin_{\varrho_{4}^{-}}^{-}\left(x\right),\\ J^{\mathrm{IV},+}\left(X_{1}\left(x\right),\ldots ,X_{n}\left(x\right)\right) & =\sin_{\varrho_{4}^{+}}^{+}\left(x\right)\sin_{\varrho_{4}^{+}}^{+}\left(x\right). \end{array}$$

In the case of polynomials $P_{k}^{\mathrm{II},-}$, the following polynomial function is introduced,

(67) $$J^{\mathrm{II},-}\left(X_{1},\ldots ,X_{n}\right)=\sin_{\varrho_{2}^{-}}^{-}\left(x\right)\sin_{\varrho_{2}^{-}}^{-}\left(x\right)={\left(\frac{J}{c}\right)}^{2}.$$

Due to Corollary 1, all four Functions (66) and (67) do not vanish in the interior of $F({\tilde{S}}_{n}^{\mathrm{aff}})$. The Equalities (14)–(17) imply that $J^{2}$ is divisible by $J^{\mathrm{II},\pm }$ and $J^{\mathrm{IV},\pm }$. The final weights $w^{\mathrm{II},\pm }$ and $w^{\mathrm{IV},\pm }$ in the continuous orthogonality relations are given for $x\in F{\left({\tilde{S}}_{n}^{\mathrm{aff}}\right)}^{\circ }$ by

(68) $$w^{\mathrm{II},\pm }=\frac{J^{\mathrm{II},\pm }}{J},\;w^{\mathrm{IV},\pm }=\frac{J^{\mathrm{IV},\pm }}{J}.$$

**Example** **5.** *For n=3, the Jacobian J in the polynomial variables is of the form*

(69) $$\begin{array}{cc} J\left(X_{1},X_{2},X_{3}\right)= & \sqrt{\pi^{6}\left(-8X_{2}^{3}+X_{1}^{2}X_{2}^{2}-12X_{3}^{2}+12X_{1}X_{2}X_{3}-\frac{4}{3}X_{1}^{3}X_{3}\right)}\\ & \times \sqrt{\left(X_{3}+3X_{2}+3X_{1}+6\right)\left(-X_{3}+3X_{2}-3X_{1}+6\right)}. \end{array}$$

*The explicit formulas of the polynomials $J^{\mathrm{II},\pm }$ and $J^{\mathrm{II},\pm }$ are given by*

(70) $$\begin{array}{cc} J^{\mathrm{II},-}\left(X_{1},X_{2},X_{3}\right)= & \frac{1}{9}\left(-8X_{2}^{3}+X_{1}^{2}X_{2}^{2}-12X_{3}^{2}+12X_{1}X_{2}X_{3}-\frac{4}{3}X_{1}^{3}X_{3}\right)\\ & \times \left(X_{3}+3X_{2}+3X_{1}+6\right)\left(-X_{3}+3X_{2}-3X_{1}+6\right),\\ J^{\mathrm{II},+}\left(X_{1},X_{2},X_{3}\right)= & \left(X_{3}+3X_{2}+3X_{1}+6\right)\left(-X_{3}+3X_{2}-3X_{1}+6\right),\\ J^{\mathrm{IV},-}\left(X_{1},X_{2},X_{3}\right)= & \frac{1}{12}\left(-8X_{2}^{3}+X_{1}^{2}X_{2}^{2}-12X_{3}^{2}+12X_{1}X_{2}X_{3}-\frac{4}{3}X_{1}^{3}X_{3}\right)\\ & \times \left(-X_{3}+3X_{2}-3X_{1}+6\right),\\ J^{\mathrm{IV},+}\left(X_{1},X_{2},X_{3}\right)= & \frac{3}{4}\left(-X_{3}+3X_{2}-3X_{1}+6\right). \end{array}$$

**Proposition** **3.** *The polynomials $P_{k}^{\mathrm{II},\pm }$ and $P_{k}^{\mathrm{IV},\pm }$ , $k\in P^{+}$, form within each family an orthogonal polynomial sequence on the integration domain $F({\tilde{S}}_{n}^{\mathrm{aff}})$ given by*

(71) $$F\left({\tilde{S}}_{n}^{\mathrm{aff}}\right)=\left\{\left(X_{1}\left(x\right),\ldots ,X_{n}\left(x\right)\right)\in R^{n}\;|\;x\in F\left({\tilde{S}}_{n}^{\mathrm{aff}}\right)\right\}$$

*and with respect to the weights $w^{\mathrm{II},\pm }$ and $w^{\mathrm{IV},\pm }$. For $X=(X_{1},\cdots ,X_{n})$, $dX=dX_{1}\cdots dX_{n}$ and $k,k^{\prime }\in P^{+}$, the polynomial orthogonality relations are of the explicit form*

(72) $$\begin{array}{ccc} \int_{F({\tilde{S}}_{n}^{\mathrm{aff}})}P_{k}^{\mathrm{II},-}\left(X\right)P_{k^{\prime }}^{\mathrm{II},-}\left(X\right)w^{\mathrm{II},-}\left(X\right)\;dX & \;=\; & 2^{-n}\delta_{kk^{\prime }},\\ \int_{F({\tilde{S}}_{n}^{\mathrm{aff}})}P_{k}^{\mathrm{II},+}\left(X\right)P_{k^{\prime }}^{\mathrm{II},+}\left(X\right)w^{\mathrm{II},+}\left(X\right)\;dX & \;=\; & 2^{-n}H_{k}\delta_{kk^{\prime }},\\ \int_{F({\tilde{S}}_{n}^{\mathrm{aff}})}P_{k}^{\mathrm{IV},-}\left(X\right)P_{k^{\prime }}^{\mathrm{IV},-}\left(X\right)w^{\mathrm{IV},-}\left(X\right)\;dX & \;=\; & 2^{-n}\delta_{kk^{\prime }},\\ \int_{F({\tilde{S}}_{n}^{\mathrm{aff}})}P_{k}^{\mathrm{IV},+}\left(X\right)P_{k^{\prime }}^{\mathrm{IV},+}\left(X\right)w^{\mathrm{IV},+}\left(X\right)\;dX & \;=\; & 2^{-n}H_{k}\delta_{kk^{\prime }}. \end{array}$$

**Proof.**  The correspondence between the domain $F({\tilde{S}}_{n}^{\mathrm{aff}})$ and $F({\tilde{S}}_{n}^{\mathrm{aff}})$ given by

(73) $$\varphi :\;x\in F\left({\tilde{S}}_{n}^{\mathrm{aff}}\right)\to \left(X_{1}\left(x\right),\ldots ,X_{n}\left(x\right)\right)\in F\left({\tilde{S}}_{n}^{\mathrm{aff}}\right)$$

is proved to be one-to-one in [3]. Therefore, the corresponding change of variables is applicable on the integration Formulas (26)–(29). □

**Corollary** **2.** *The polynomials $P_{k}^{\mathrm{II},\pm }$ and $P_{k}^{\mathrm{IV},\pm }$, $k\in P^{+}$ form an orthogonal basis of all polynomials f,g∈R[X] of n variables with respect to the scalar product defined by*

(74) $${\langle f,g\rangle }_{w}=\int_{F({\tilde{S}}_{n}^{\mathrm{aff}})}f\left(X\right)g\left(X\right)w\left(X\right)\;dX,\;w\in \left\{w^{\mathrm{II},\pm },w^{\mathrm{IV},\pm }\right\}.$$

**Proof.**  Proposition 4.1 in [3] grants that the number of polynomials (51) of degree *d* is equal to the number of monomials of *n* variables of degree *d*. □

### 4.3. Cubature Formulas

The main objective of cubature formulas is to estimate weighted integrals of multivariate integrable functions f(Y), $Y\in R^{n}$, over an integration domain $\Omega \subset R^{n}$ with a weight *w* by linear combinations of function values at a suitable finite set of points $\Omega_{n}\subset \Omega$,

(75) $$\int_{\Omega }f\left(Y\right)w\left(Y\right)dY\approx \sum_{Y\in \Omega_{n}}c_{Y}f\left(Y\right).$$

The points from $\Omega_{n}$ are generally called nodes. It is required that the cubature formulas hold exactly for polynomials up to a certain degree. The optimal cubature formulas in a sense of minimal number of points on which functions need to be evaluated are called Gaussian. In the following, the integrals over the domain $F({\tilde{S}}_{n}^{\mathrm{aff}})$ with weights $w\in \{w^{\mathrm{II},\pm },w^{\mathrm{IV},\pm }\}$ are replaced by finite summing.

The finite sets $F_{N}^{★,\pm }$, ★∈I,II,…,VIII of generalized Chebyshev nodes, on which cubature formulas are evaluated, are connected to the points in the discrete sets $F_{N}^{★,\pm }$ from Table 1 by the φ-transform (73),

(76) $$F_{N}^{★,\pm }=\left\{\varphi \left(s\right)\;|\;s\in F_{N}^{★,\pm }\right\}.$$

Recall from [3] that the φ-transform is one-to-one correspondence between $F_{N}^{★,\pm }$ and $F_{N}^{★,\pm }$ and therefore, for each $Y\in F_{N}^{★,\pm }$ there exists a unique $s\in F_{N}^{★,\pm }$ such that Y=φ(s). Thus, the following three weight symbols are well-defined,

(77) $$H_{Y}=H_{s},\;E_{Y}=\varepsilon_{s},\;{\tilde{E}}_{Y}={\tilde{\varepsilon }}_{s}.$$

Each family of orthogonal polynomials (51) gives rise to four different cubature formulas. The formulas related to $P^{\mathrm{II},-}$ are derived from AMDST of type I,II,V and VI whereas the formulas related to $P^{\mathrm{II},+}$ arise from the symmetric discrete transforms of the same types. Similarly, the formulas related to $P^{\mathrm{IV},-}$ are deduced from AMDST of type III,IV,VII and VIII and the formulas related to $P^{\mathrm{IV},+}$ arise from the symmetric discrete transforms of the same types.

**Theorem** **1.**

*1.*  *For N∈N, N≥n and any polynomial f(Y) of degree at most 2(N−n)+1, the following formula holds exactly,*
  (78) $$\int_{F({\tilde{S}}_{n}^{\mathrm{aff}})}f\left(Y\right)w^{\mathrm{II},-}\left(Y\right)\;dY={\left(\frac{1}{N+1}\right)}^{n}\sum_{Y\in F_{N}^{I,-}}f\left(Y\right)J^{\mathrm{II},-}\left(Y\right).$$
*2.*  *For N∈N, N≥n+1 and any polynomial f(Y) of degree at most 2(N−n)−1, the following formula holds exactly,*
  (79) $$\int_{F({\tilde{S}}_{n}^{\mathrm{aff}})}f\left(Y\right)w^{\mathrm{II},-}\left(Y\right)\;dY={\left(\frac{1}{N}\right)}^{n}\sum_{Y\in F_{N}^{\mathrm{II},-}}f\left(Y\right)J^{\mathrm{II},-}\left(Y\right).$$
*3.*  *For N∈N, N≥n and any polynomial f(Y) of degree at most 2(N−n), the following formulas hold exactly,*
  (80) $$\begin{array}{ccc} \int_{F({\tilde{S}}_{n}^{\mathrm{aff}})}f\left(Y\right)w^{\mathrm{II},-}\left(Y\right)\;dY & = & {\left(\frac{2}{2N+1}\right)}^{n}\sum_{Y\in F_{N}^{V,-}}f\left(Y\right)J^{\mathrm{II},-}\left(Y\right),\\ \int_{F({\tilde{S}}_{n}^{\mathrm{aff}})}f\left(Y\right)w^{\mathrm{II},-}\left(Y\right)\;dY & = & {\left(\frac{2}{2N+1}\right)}^{n}\sum_{Y\in F_{N}^{\mathrm{VI},-}}f\left(Y\right)J^{\mathrm{II},-}\left(Y\right). \end{array}$$

**Proof.**  The linearity of Equation (78) implies that it is sufficient to consider f(Y) in form of a monomial of degree at most 2(N−n)+1. Any such monomial is expressible as a product of a monomial p(Y) of degree not exceeding N−n+1 and a monomial q(Y) of degree at most N−n. From Proposition 2 and Corollary 2 it follows that the monomials *p* and *q* are expressible as linear combinations of polynomials $P_{k}^{\mathrm{II},-}$ with $k_{1}\leq N-n+1$ and $P_{k^{\prime }}^{\mathrm{II},-}$ with $k_{1}^{\prime }\leq N-n$, respectively. From the transform AMDST-I, given by (40), and Remark 1, these polynomials satisfy the following discrete orthogonality relation,

(81) $${\left(\frac{1}{N+1}\right)}^{n}\sum_{Y\in F_{N}^{I,-}}P_{k}^{\mathrm{II},-}\left(Y\right)P_{k^{\prime }}^{\mathrm{II},-}\left(Y\right)J^{\mathrm{II},-}\left(Y\right)=2^{-n}\delta_{kk^{\prime }}.$$

Comparing (72) and (81), the cubature Formula (78) is derived. Other cubature formulas are deduced similarly from AMDST of type II, V and VI. □

**Theorem** **2.**

*1.*  *For N∈N and any polynomial f(Y) of degree at most 2N−1, the following formula holds exactly,*
  (82) $$\int_{F({\tilde{S}}_{n}^{\mathrm{aff}})}f\left(Y\right)w^{\mathrm{II},+}\left(Y\right)\;dY={\left(\frac{1}{N+1}\right)}^{n}\sum_{Y\in F_{N}^{I,+}}H_{Y}^{-1}f\left(Y\right)J^{\mathrm{II},+}\left(Y\right).$$
*2.*  *For N∈N, N>1 and any polynomial f(Y) of degree at most 2N−3, the following formula holds exactly,*
  (83) $$\int_{F({\tilde{S}}_{n}^{\mathrm{aff}})}f\left(Y\right)w^{\mathrm{II},+}\left(Y\right)\;dY={\left(\frac{1}{N}\right)}^{n}\sum_{Y\in F_{N}^{\mathrm{II},+}}H_{Y}^{-1}f\left(Y\right)J^{\mathrm{II},+}\left(Y\right).$$
*3.*  *For N∈N and any polynomial f(Y) of degree at most 2N−2, the following formulas hold exactly,*
  (84) $$\begin{array}{ccc} \int_{F({\tilde{S}}_{n}^{\mathrm{aff}})}f\left(Y\right)w^{\mathrm{II},+}\left(Y\right)\;dY & = & {\left(\frac{2}{2N+1}\right)}^{n}\sum_{Y\in F_{N}^{V,+}}H_{Y}^{-1}f\left(Y\right)J^{\mathrm{II},+}\left(Y\right),\\ \int_{F({\tilde{S}}_{n}^{\mathrm{aff}})}f\left(Y\right)w^{\mathrm{II},+}\left(Y\right)\;dY & = & {\left(\frac{2}{2N+1}\right)}^{n}\sum_{Y\in F_{N}^{\mathrm{VI},+}}H_{Y}^{-1}f\left(Y\right)J^{\mathrm{II},+}\left(Y\right). \end{array}$$

**Theorem** **3.**

*1.*  *For N∈N, N≥n and any polynomial f(Y) of degree at most 2(N−n)+1, the following formula holds exactly,*
  (85) $$\int_{F({\tilde{S}}_{n}^{\mathrm{aff}})}f\left(Y\right)w^{\mathrm{IV},-}\left(Y\right)\;dY={\left(\frac{2}{2N+1}\right)}^{n}\sum_{Y\in F_{N}^{\mathrm{VII},-}}f\left(Y\right)J^{\mathrm{IV},-}\left(Y\right).$$
*2.*  *For N∈N, N≥n and any polynomial f(Y) of degree at most 2(N−n), the following formulas hold exactly,*
  (86) $$\begin{array}{ccc} \int_{F({\tilde{S}}_{n}^{\mathrm{aff}})}f\left(Y\right)w^{\mathrm{IV},-}\left(Y\right)\;dY & = & {\left(\frac{1}{N}\right)}^{n}\sum_{Y\in F_{N}^{\mathrm{III},-}}E_{Y}f\left(Y\right)J^{\mathrm{IV},-}\left(Y\right),\\ \int_{F({\tilde{S}}_{n}^{\mathrm{aff}})}f\left(Y\right)w^{\mathrm{IV},-}\left(Y\right)\;dY & = & {\left(\frac{1}{N}\right)}^{n}\sum_{Y\in F_{N}^{\mathrm{IV},-}}f\left(Y\right)J^{\mathrm{IV},-}\left(Y\right). \end{array}$$
*3.*  *For N∈N, N≥n+1 and any polynomial f(Y) of degree at most 2(N−n)−1, the following formula holds exactly,*
  (87) $$\int_{F({\tilde{S}}_{n}^{\mathrm{aff}})}f\left(Y\right)w^{\mathrm{IV},-}\left(Y\right)\;dY={\left(\frac{2}{2N-1}\right)}^{n}\sum_{Y\in F_{N}^{\mathrm{VIII},-}}{\tilde{E}}_{Y}f\left(Y\right)J^{\mathrm{IV},-}\left(Y\right).$$

**Theorem** **4.**

*1.*  *For N∈N and any polynomial f(Y) of degree at most 2N−1, the following formula holds exactly,*
  (88) $$\int_{F({\tilde{S}}_{n}^{\mathrm{aff}})}f\left(Y\right)w^{\mathrm{IV},+}\left(Y\right)\;dY={\left(\frac{2}{2N+1}\right)}^{n}\sum_{Y\in F_{N}^{\mathrm{VII},+}}H_{Y}^{-1}f\left(Y\right)J^{\mathrm{IV},+}\left(Y\right).$$
*2.*  *For N∈N and any polynomial f(Y) of degree at most 2N−2, the following formulas hold exactly,*
  (89) $$\begin{array}{ccc} \int_{F({\tilde{S}}_{n}^{\mathrm{aff}})}f\left(Y\right)w^{\mathrm{IV},+}\left(Y\right)\;dY & = & {\left(\frac{1}{N}\right)}^{n}\sum_{Y\in F_{N}^{\mathrm{III},+}}E_{Y}H_{Y}^{-1}f\left(Y\right)J^{\mathrm{IV},+}\left(Y\right),\\ \int_{F({\tilde{S}}_{n}^{\mathrm{aff}})}f\left(Y\right)w^{\mathrm{IV},+}\left(Y\right)\;dY & = & {\left(\frac{1}{N}\right)}^{n}\sum_{Y\in F_{N}^{\mathrm{IV},+}}H_{Y}^{-1}f\left(Y\right)J^{\mathrm{IV},+}\left(Y\right). \end{array}$$
*3.*  *For N∈N, N>1 and any polynomial f(Y) of degree at most 2N−3, the following formulas hold exactly,*
  (90) $$\int_{F({\tilde{S}}_{n}^{\mathrm{aff}})}f\left(Y\right)w^{\mathrm{IV},+}\left(Y\right)\;dY={\left(\frac{2}{2N-1}\right)}^{n}\sum_{Y\in F_{N}^{\mathrm{VIII},+}}{\tilde{E}}_{Y}H_{Y}^{-1}f\left(Y\right)J^{\mathrm{IV},+}\left(Y\right).$$

### 4.4. Gaussian Cubature Formulas

**Theorem** **5.** *The cubature Formulas* (78), (82), (85) *and* (88) *are optimal Gaussian cubature formulas. Furthermore, it holds that*

*1.*  *the orthogonal polynomials $P_{k}^{\mathrm{II},-}$ of degree $k_{1}=N-n+1$ vanish for all nodes $F_{N}^{I,-}$,*
*2.*  *the orthogonal polynomials $P_{k}^{\mathrm{II},+}$ of degree $k_{1}=N$ vanish for all nodes $F_{N}^{I,+}$,*
*3.*  *the orthogonal polynomials $P_{k}^{\mathrm{IV},-}$ of degree $k_{1}=N-n+1$ vanish for all nodes $F_{N}^{\mathrm{VII},-}$,*
*4.*  *the orthogonal polynomials $P_{k}^{\mathrm{IV},+}$ of degree $k_{1}=N$ vanish for nodes $F_{N}^{\mathrm{VII},+}$.*

**Proof.**  The number of nodes in the set $F_{N}^{I,-}$ is equal to the number of points in $F_{N}^{I,-}$ since φ is injective on $F_{N}^{I,-}$. The definition of $F_{N}^{I,-}$ in Table 1 and definition (37) imply that

(91) $$|F_{N}^{I,-}\left|=\right|D_{1,N}^{-}|$$

and the cardinality of these sets corresponds to the number of polynomials $P_{k}^{\mathrm{II},-}$ of degree N−n. Therefore, the cubature Formula (78) is Gaussian. Similar counting arguments prove that the remaining listed formulas are also Gaussian. The fact that the nodes are common zeros of the corresponding polynomials of a specific degree follows directly from definition (51) and Remarks 1 and 2. □

The Gaussian cubatures (78), (82), (85) and (88) are special cases of the general cubature Formulas (1) and (2) from [22], where the values of integrals

(92) $$\int f\left(u\right){\left[\Delta \left(u\right)\right]}^{\pm \frac{1}{2}}\;d\nu \left(u\right)$$

are studied. The variables $u_{i}$ are connected to the elementary symmetric functions by relation

(93) $$u_{i}=u_{i}\left(y_{1},\cdots ,y_{n}\right)=\sum_{1\leq k_{1}<\cdots <k_{i}\leq n}y_{k_{1}}\cdots y_{k_{i}}$$

and coincide, up to a multiplication by a constant, with the current polynomial variables $X_{i}$,

(94) $$u_{i}=\frac{X_{i}}{(n-i)!i!}.$$

The measures w(X)dX, $w\in \{w^{\mathrm{II},\pm },w^{\mathrm{IV},\pm }\}$, given by (68), correspond to the special choices of the positive measure ${\left[\Delta \left(u\right)\right]}^{\pm \frac{1}{2}}\;d\nu \left(u\right)$ of the form μ(u)du with

(95) $$\mu \left(u\right)=\prod_{i=1}^{n}{\left(1-y_{i}\right)}^{\alpha }{\left(1+y_{i}\right)}^{\beta }{\left(\prod_{1\leq i<j\leq n}{\left(y_{i}-y_{j}\right)}^{2}\right)}^{\gamma },\;$$

and

(96) $$\alpha ,\beta ,\gamma \in \left\{\pm \frac{1}{2}\right\},\;-1<y_{1}<\cdots <y_{n}<1.$$

In particular, the parameters α,β and γ have the following values depending on the chosen family of polynomials,

(97) $$\begin{array}{ccc} P_{k}^{\mathrm{II},-}: & \; & \alpha =\beta =\gamma =\frac{1}{2},\\ P_{k}^{\mathrm{II},+}: & \; & \alpha =\beta =\frac{1}{2},\;\gamma =-\frac{1}{2},\\ P_{k}^{\mathrm{IV},-}: & \; & \alpha =\gamma =\frac{1}{2},\;\beta =-\frac{1}{2},\\ P_{k}^{\mathrm{IV},+}: & \; & \alpha =\frac{1}{2},\;\beta =\gamma =-\frac{1}{2}. \end{array}$$

The cubature Formulas (78), (82), (85) and (88) together with the Gaussian cubatures from [3] form the set of cubatures with all possible values of parameters in (95).

## 5. Conclusions

- The present fully explicit expression of the cubature rules allows straightforward implementation of the numerical integration and approximation methods. Compared to the abstract variables of the symmetric polynomials (93) from [22], the additional relation (49) established via the fundamental symmetric cosine function connects directly, like in the classical Chebyshev polynomials, the underlying lattice with the generalized Chebyshev nodes. The antisymmetric discrete sine transforms from Table 1 are special cases of the discrete transforms derived in [14] from generalized Schur polynomials associated with Bernstein-Szegö polynomials and parametrized by $\left(a_{-},b_{-}\right)\in \left\{\left(0,0\right),\left(0,-1\right)\right\}$. On the other hand, the symmetric discrete sine transforms from Table 1 extend the set of discrete transforms connected to the Chebyshev polynomials of the second and fourth kind.
- The symmetry group of the (anti)symmetric sine functions ${\left(Z/2Z\right)}^{n}⋊S_{n}$ is isomorphic to the Weyl groups of the classical series of the simple Lie algebras $B_{n}$ and $C_{n}$. The correspondence between the (anti)symmetric sine and cosine functions and the four types of the Weyl orbit functions is explicitly developed in [9]. The present point sets of the discrete (anti)symmetric sine transforms and the generalized Chebyshev nodes differ from the weight and dual weight lattice point sets on which the discrete transforms and cubature rules of the Weyl orbit functions are formulated. The topology of the current point sets is, however, similar for some cases to the root lattices of the series $B_{n}$ and $C_{n}$ and the explicit formulation of the comparison poses an open problem.
- The Lebesgue constant estimates of the polynomial cubatures and integral error estimates for the interpolation formulas together with criteria for the convergence of the polynomial series deserve further study. The developed cubature formulas as well as the rules from [3,8] reveal that the shifted lattice transforms carry high capacity to produce cubature formulas of Gaussian type. Versions of the Clenshaw–Curtis methods of numerical integration [36], developed for the $C_{2}$ and $A_{2}$ root systems in [37,38], also need to be further investigated. The formation of the hyperinterpolation methods [39,40], which straightforwardly employ the standard polynomial cubature rules, poses an open problem for the presented cubature rules.
- The existence and explicit forms of generating functions for the related Weyl group polynomials, developed in [41,42], further increase the relevance of the presented Chebyshev polynomial methods. The generating functions form a powerful tool for investigating symmetries and parity relations of the generated orthogonal polynomials and represent practical tool for efficient computer implementation and handling of the generated polynomials. The recurrence relations algorithms for the calculation of the trivariate polynomials are potentially superseded by explicit evaluation formulas derived from the generating functions. The form of the generating functions and the explicit evaluation formulas for the current polynomials pose open problems.

## Figures and Tables

**Table 1:** The sets of labels $D_{N}^{★,-},D_{N}^{★,+}$ and sets of points $F_{N}^{★,-},F_{N}^{★,+}$ together with the weights $\varepsilon^{★}$ and normalization coefficients $h^{★}$ are specified for each type ★∈{I,II,⋯,VIII} of antisymmetric generalizations of discrete sine transforms (DSTs) (AMDST), and symmetric generalizations of DSTs (SMDST), respectively.

★	$D_{N}^{★,-}$	$F_{N}^{★,-}$	$D_{N}^{★,+}$	$F_{N}^{★,+}$	$h_{k}^{★}$	$\varepsilon_{s}^{★}$
I	$D_{1,N}^{-}$	$\frac{1}{N+1}D_{1,N}^{-}$	$D_{1,N}^{+}$	$\frac{1}{N+1}D_{1,N}^{+}$	${\left(\frac{N+1}{2}\right)}^{n}$	1
II	$D_{1,N}^{-}$	$\frac{1}{N}D_{1,N,\varrho }^{-}$	$D_{1,N}^{+}$	$\frac{1}{N}D_{1,N,\varrho }^{+}$	$d_{k}^{-1}{\left(\frac{N}{2}\right)}^{n}$	1
III	$D_{1,N,\varrho }^{-}$	$\frac{1}{N}D_{1,N}^{-}$	$D_{1,N,\varrho }^{+}$	$\frac{1}{N}D_{1,N}^{+}$	${\left(\frac{N}{2}\right)}^{n}$	$\varepsilon_{s}$
IV	$D_{1,N,\varrho }^{-}$	$\frac{1}{N}D_{1,N,\varrho }^{-}$	$D_{1,N,\varrho }^{+}$	$\frac{1}{N}D_{1,N,\varrho }^{+}$	${\left(\frac{N}{2}\right)}^{n}$	1
V	$D_{1,N}^{-}$	$\frac{2}{2N+1}D_{1,N}^{-}$	$D_{1,N}^{+}$	$\frac{2}{2N+1}D_{1,N}^{+}$	${\left(\frac{2N+1}{4}\right)}^{n}$	1
VI	$D_{1,N}^{-}$	$\frac{2}{2N+1}D_{1,N,\varrho }^{-}$	$D_{1,N}^{+}$	$\frac{2}{2N+1}D_{1,N,\varrho }^{+}$	${\left(\frac{2N+1}{4}\right)}^{n}$	1
VII	$D_{1,N,\varrho }^{-}$	$\frac{2}{2N+1}D_{1,N}^{-}$	$D_{1,N,\varrho }^{+}$	$\frac{2}{2N+1}D_{1,N}^{+}$	${\left(\frac{2N+1}{4}\right)}^{n}$	1
VIII	$D_{1,N,\varrho }^{-}$	$\frac{2}{2N-1}D_{1,N,\varrho }^{-}$	$D_{1,N,\varrho }^{+}$	$\frac{2}{2N-1}D_{1,N,\varrho }^{+}$	${\tilde{d}}_{k}^{-1}{\left(\frac{2N-1}{4}\right)}^{n}$	${\tilde{\varepsilon }}_{s}$

**Table 2:** Integral error estimates of the polynomial approximations of the model function (47) by $\psi_{N}^{\mathrm{II},\pm },\psi_{N}^{\mathrm{III},\pm },\psi_{N}^{\mathrm{VI},\pm }$ and $\psi_{N}^{\mathrm{VII},\pm }$ for N=7,12,17,22,27.

***N***	$\int_{F({\tilde{S}}_{n}^{\mathrm{aff}})}{\left|f-\psi_{N}^{\mathrm{II},-}\right|}^{2}$	$\int_{F({\tilde{S}}_{n}^{\mathrm{aff}})}{\left|f-\psi_{N}^{\mathrm{III},-}\right|}^{2}$	$\int_{F({\tilde{S}}_{n}^{\mathrm{aff}})}{\left|f-\psi_{N}^{\mathrm{VI},-}\right|}^{2}$	$\int_{F({\tilde{S}}_{n}^{\mathrm{aff}})}{\left|f-\psi_{N}^{\mathrm{VII},-}\right|}^{2}$
7	$144,637\times 10^{-6}$	$204,640\times 10^{-6}$	$123,618\times 10^{-6}$	$121,379\times 10^{-6}$
12	$8850\times 10^{-6}$	$11,715\times 10^{-6}$	$7540\times 10^{-6}$	$5554\times 10^{-6}$
17	$238\times 10^{-6}$	$358\times 10^{-6}$	$113\times 10^{-6}$	$96\times 10^{-6}$
22	$21\times 10^{-6}$	$21\times 10^{-6}$	$20\times 10^{-6}$	$11\times 10^{-6}$
27	$18\times 10^{-6}$	$17\times 10^{-6}$	$2\times 10^{-6}$	$7\times 10^{-6}$
N	$\int_{F({\tilde{S}}_{n}^{\mathrm{aff}})}{\left|f-\psi_{N}^{\mathrm{II},+}\right|}^{2}$	$\int_{F({\tilde{S}}_{n}^{\mathrm{aff}})}{\left|f-\psi_{N}^{\mathrm{III},+}\right|}^{2}$	$\int_{F({\tilde{S}}_{n}^{\mathrm{aff}})}{\left|f-\psi_{N}^{\mathrm{VI},+}\right|}^{2}$	$\int_{F({\tilde{S}}_{n}^{\mathrm{aff}})}{\left|f-\psi_{N}^{\mathrm{VII},+}\right|}^{2}$
7	$146,367\times 10^{-6}$	$146,252\times 10^{-6}$	$190,670\times 10^{-6}$	$158,619\times 10^{-6}$
12	$5639\times 10^{-6}$	$11,502\times 10^{-6}$	$9971\times 10^{-6}$	$8792\times 10^{-6}$
17	$192\times 10^{-6}$	$297\times 10^{-6}$	$238\times 10^{-6}$	$191\times 10^{-6}$
22	$11\times 10^{-6}$	$14\times 10^{-6}$	$20\times 10^{-6}$	$11\times 10^{-6}$
27	$8\times 10^{-6}$	$7\times 10^{-6}$	$16\times 10^{-6}$	$6\times 10^{-6}$

**Table 3:** The coefficients of the polynomials $P_{\left(k_{1},k_{2},k_{3}\right)}^{\mathrm{II},\pm }$ with $k_{1}\leq 2$ and $k_{1}+k_{2}+k_{3}$ even.

$P_{\left(k_{1},k_{2},k_{3}\right)}^{\mathrm{II},-}$	1	$X_{2}$	$X_{1}^{2}$	$X_{1}X_{3}$	$X_{2}^{2}$	$X_{3}^{2}$	$P_{\left(k_{1},k_{2},k_{3}\right)}^{\mathrm{II},+}$	1	$X_{2}$	$X_{1}^{2}$	$X_{1}X_{3}$	$X_{2}^{2}$	$X_{3}^{2}$
$P_{\left(0,0,0\right)}^{\mathrm{II},-}$	1						$P_{\left(0,0,0\right)}^{\mathrm{II},+}$	1					
$P_{\left(1,1,0\right)}^{\mathrm{II},-}$	2	2					$P_{\left(1,1,0\right)}^{\mathrm{II},+}$	0	$\frac{2}{3}$				
$P_{\left(2,0,0\right)}^{\mathrm{II},-}$	−3	−2	1				$P_{\left(2,0,0\right)}^{\mathrm{II},+}$	−1	$-\frac{4}{3}$	$\frac{1}{3}$			
$P_{\left(2,1,1\right)}^{\mathrm{II},-}$	−2	−2	1	$\frac{4}{3}$			$P_{\left(2,1,1\right)}^{\mathrm{II},+}$	0	$-\frac{2}{3}$	0	$\frac{4}{9}$		
$P_{\left(2,2,0\right)}^{\mathrm{II},-}$	6	10	−2	$-\frac{4}{3}$	4		$P_{\left(2,2,0\right)}^{\mathrm{II},+}$	1	$\frac{8}{3}$	$-\frac{2}{3}$	$-\frac{8}{9}$	$\frac{4}{3}$	
$P_{\left(2,2,2\right)}^{\mathrm{II},-}$	−4	−8	2	$\frac{12}{3}$	−4	$\frac{16}{9}$	$P_{\left(2,2,2\right)}^{\mathrm{II},+}$	−1	−4	1	$\frac{8}{3}$	−4	$\frac{16}{9}$

**Table 4:** The coefficients of the polynomials $P_{\left(k_{1},k_{2},k_{3}\right)}^{\mathrm{II},\pm }$ with $k_{1}\leq 2$ and $k_{1}+k_{2}+k_{3}$ odd.

$P_{\left(k_{1},k_{2},k_{3}\right)}^{\mathrm{II},-}$	$X_{1}$	$X_{3}$	$X_{1}X_{2}$	$X_{2}X_{3}$	$P_{\left(k_{1},k_{2},k_{3}\right)}^{\mathrm{II},+}$	$X_{1}$	$X_{3}$	$X_{1}X_{2}$	$X_{2}X_{3}$
$P_{\left(1,0,0\right)}^{\mathrm{II},-}$	1				$P_{\left(1,0,0\right)}^{\mathrm{II},+}$	$\frac{1}{3}$			
$P_{\left(1,1,1\right)}^{\mathrm{II},-}$	1	$\frac{4}{3}$			$P_{\left(1,1,1\right)}^{\mathrm{II},+}$	0	$\frac{4}{3}$		
$P_{\left(2,1,0\right)}^{\mathrm{II},-}$	0	$-\frac{4}{3}$	2		$P_{\left(2,1,0\right)}^{\mathrm{II},+}$	$-\frac{1}{3}$	$-\frac{2}{3}$	$\frac{1}{3}$	
$P_{\left(2,2,1\right)}^{\mathrm{II},-}$	1	4	0	$\frac{8}{3}$	$P_{\left(2,2,1\right)}^{\mathrm{II},+}$	$\frac{1}{3}$	$\frac{4}{3}$	$-\frac{2}{3}$	$\frac{8}{9}$

**Table 5:** The coefficients of the polynomials $P_{\left(k_{1},k_{2},k_{3}\right)}^{\mathrm{IV},-}$ with $k_{1}\leq 2$.

$P_{\left(k_{1},k_{2},k_{3}\right)}^{\mathrm{IV},-}$	1	$X_{1}$	$X_{2}$	$X_{3}$	$X_{1}^{2}$	$X_{1}X_{2}$	$X_{1}X_{3}$	$X_{2}^{2}$	$X_{2}X_{3}$	$X_{3}^{2}$
$P_{\left(0,0,0\right)}^{\mathrm{IV},-}$	1									
$P_{\left(1,0,0\right)}^{\mathrm{IV},-}$	1	1								
$P_{\left(1,1,0\right)}^{\mathrm{IV},-}$	3	1	2							
$P_{\left(1,1,1\right)}^{\mathrm{IV},-}$	3	2	2	$\frac{4}{3}$						
$P_{\left(2,0,0\right)}^{\mathrm{IV},-}$	−3	1	−2	0	1					
$P_{\left(2,1,0\right)}^{\mathrm{IV},-}$	−1	1	0	$-\frac{4}{3}$	1	2				
$P_{\left(2,1,1\right)}^{\mathrm{IV},-}$	−3	2	−2	0	2	2	$\frac{4}{3}$			
$P_{\left(2,2,0\right)}^{\mathrm{IV},-}$	8	0	12	$-\frac{4}{3}$	−2	2	$-\frac{4}{3}$	4		
$P_{\left(2,2,1\right)}^{\mathrm{IV},-}$	6	2	10	4	−1	2	0	4	$\frac{8}{3}$	
$P_{\left(2,2,2\right)}^{\mathrm{IV},-}$	−6	2	−10	$\frac{16}{3}$	3	0	$\frac{16}{3}$	−4	$\frac{8}{3}$	$\frac{16}{9}$

**Table 6:** The coefficients of the polynomials $P_{\left(k_{1},k_{2},k_{3}\right)}^{\mathrm{IV},+}$ with $k_{1}\leq 2$.

$P_{\left(k_{1},k_{2},k_{3}\right)}^{\mathrm{IV},+}$	1	$X_{1}$	$X_{2}$	$X_{3}$	$X_{1}^{2}$	$X_{1}X_{2}$	$X_{1}X_{3}$	$X_{2}^{2}$	$X_{2}X_{3}$	$X_{3}^{2}$
$P_{\left(0,0,0\right)}^{\mathrm{IV},+}$	1									
$P_{\left(1,0,0\right)}^{\mathrm{IV},+}$	1	$\frac{1}{3}$								
$P_{\left(1,1,0\right)}^{\mathrm{IV},+}$	1	$\frac{2}{3}$	$\frac{2}{3}$							
$P_{\left(1,1,1\right)}^{\mathrm{IV},+}$	1	1	2	$\frac{4}{3}$						
$P_{\left(2,0,0\right)}^{\mathrm{IV},+}$	−1	$\frac{1}{3}$	$-\frac{4}{3}$	0	$\frac{1}{3}$					
$P_{\left(2,1,0\right)}^{\mathrm{IV},+}$	−1	0	$-\frac{2}{3}$	$-\frac{2}{3}$	$\frac{1}{3}$	$\frac{1}{3}$				
$P_{\left(2,1,1\right)}^{\mathrm{IV},+}$	−1	$-\frac{1}{3}$	$-\frac{2}{3}$	0	$\frac{1}{3}$	$\frac{2}{3}$	$\frac{4}{9}$			
$P_{\left(2,2,0\right)}^{\mathrm{IV},+}$	1	$-\frac{2}{3}$	$\frac{10}{3}$	$-\frac{4}{3}$	$-\frac{2}{3}$	$\frac{2}{3}$	$-\frac{8}{9}$	$\frac{4}{3}$		
$P_{\left(2,2,1\right)}^{\mathrm{IV},+}$	1	$-\frac{1}{3}$	2	$\frac{4}{3}$	$-\frac{2}{3}$	0	0	$\frac{4}{3}$	$\frac{8}{9}$	
$P_{\left(2,2,2\right)}^{\mathrm{IV},+}$	−1	1	−6	$\frac{16}{3}$	1	−2	4	−4	$\frac{8}{3}$	$\frac{16}{9}$
